# Integrating Computational and Biological Hemodynamic Approaches to Improve Modeling of Atherosclerotic Arteries

**DOI:** 10.1002/advs.202307627

**Published:** 2024-05-05

**Authors:** Thao Nhu Anne Marie Vuong, Michael Bartolf‐Kopp, Kristina Andelovic, Tomasz Jungst, Nona Farbehi, Steven G. Wise, Christopher Hayward, Michael Charles Stevens, Jelena Rnjak‐Kovacina

**Affiliations:** ^1^ Graduate School of Biomedical Engineering University of New South Wales Sydney 2052 Australia; ^2^ Department of Functional Materials in Medicine and Dentistry Institute of Functional Materials and Biofabrication (IFB) KeyLab Polymers for Medicine of the Bavarian Polymer Institute (BPI) University of Würzburg Pleicherwall 2 97070 Würzburg Germany; ^3^ Department of Orthopedics, Regenerative Medicine Center Utrecht University Medical Center Utrecht Utrecht 3584 Netherlands; ^4^ Tyree Institute of Health Engineering University of New South Wales Sydney NSW 2052 Australia; ^5^ Garvan Weizmann Center for Cellular Genomics Garvan Institute of Medical Research Sydney NSW 2010 Australia; ^6^ School of Medical Sciences University of Sydney Sydney NSW 2006 Australia; ^7^ St Vincent's Hospital Sydney Victor Chang Cardiac Research Institute Sydney 2010 Australia; ^8^ Australian Centre for NanoMedicine (ACN) University of New South Wales Sydney NSW 2052 Australia

**Keywords:** atherosclerosis, cardiovascular disease, computational modeling, haemodynamics, tissue engineering

## Abstract

Atherosclerosis is the primary cause of cardiovascular disease, resulting in mortality, elevated healthcare costs, diminished productivity, and reduced quality of life for individuals and their communities. This is exacerbated by the limited understanding of its underlying causes and limitations in current therapeutic interventions, highlighting the need for sophisticated models of atherosclerosis. This review critically evaluates the computational and biological models of atherosclerosis, focusing on the study of hemodynamics in atherosclerotic coronary arteries. Computational models account for the geometrical complexities and hemodynamics of the blood vessels and stenoses, but they fail to capture the complex biological processes involved in atherosclerosis. Different in vitro and in vivo biological models can capture aspects of the biological complexity of healthy and stenosed vessels, but rarely mimic the human anatomy and physiological hemodynamics, and require significantly more time, cost, and resources. Therefore, emerging strategies are examined that integrate computational and biological models, and the potential of advances in imaging, biofabrication, and machine learning is explored in developing more effective models of atherosclerosis.

## Graphical abstract

This review describes and critically evaluates computational and biological models of atherosclerosis, focusing on the hemodynamics of occluded coronary arteries. It examines emerging strategies that integrate computational and biological models and explores the advances in imaging, biofabrication, single‐cell omics, and machine learning for model refinement, validation, and application in discovery research, diagnostics, and drug and medical device development and testing.

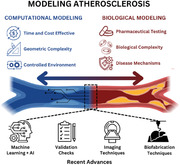

## 1 Introduction

According to the World Health Organization, cardiovascular disease is the leading cause of death globally, representing 32% of all deaths in 2019. Atherosclerosis or narrowing of arteries due to plaque buildup can affect most arteries in the body, but atherosclerosis in coronary arteries that supply the heart muscle with oxygen and nutrients is the leading cause of death in developed countries. While a range of in silico, in vitro, and in vivo models of atherosclerosis exist and are routinely used in discovery research and pre‐clinical testing of drugs and medical devices, no single model captures all aspects of the human anatomy, physiology, and pathology involved in atherosclerosis. This review offers an overview of computational and biological models of atherosclerosis, with the goal of enhancing the reader's understanding of different types of models in the field and emphasizing the need for increased and improved model validation. We propose that validation can be achieved through a synergistic approach, combining in silico models with biological in vitro models, alongside advancements in imaging and omics technology. The review critically assesses the existing gap between computational and biological models to highlight the importance of bridging this divide for a more holistic understanding of atherosclerosis, suggesting potential avenues for future research and development.

## 2 Pathophysiology of Atherosclerosis

**Figure 1:**
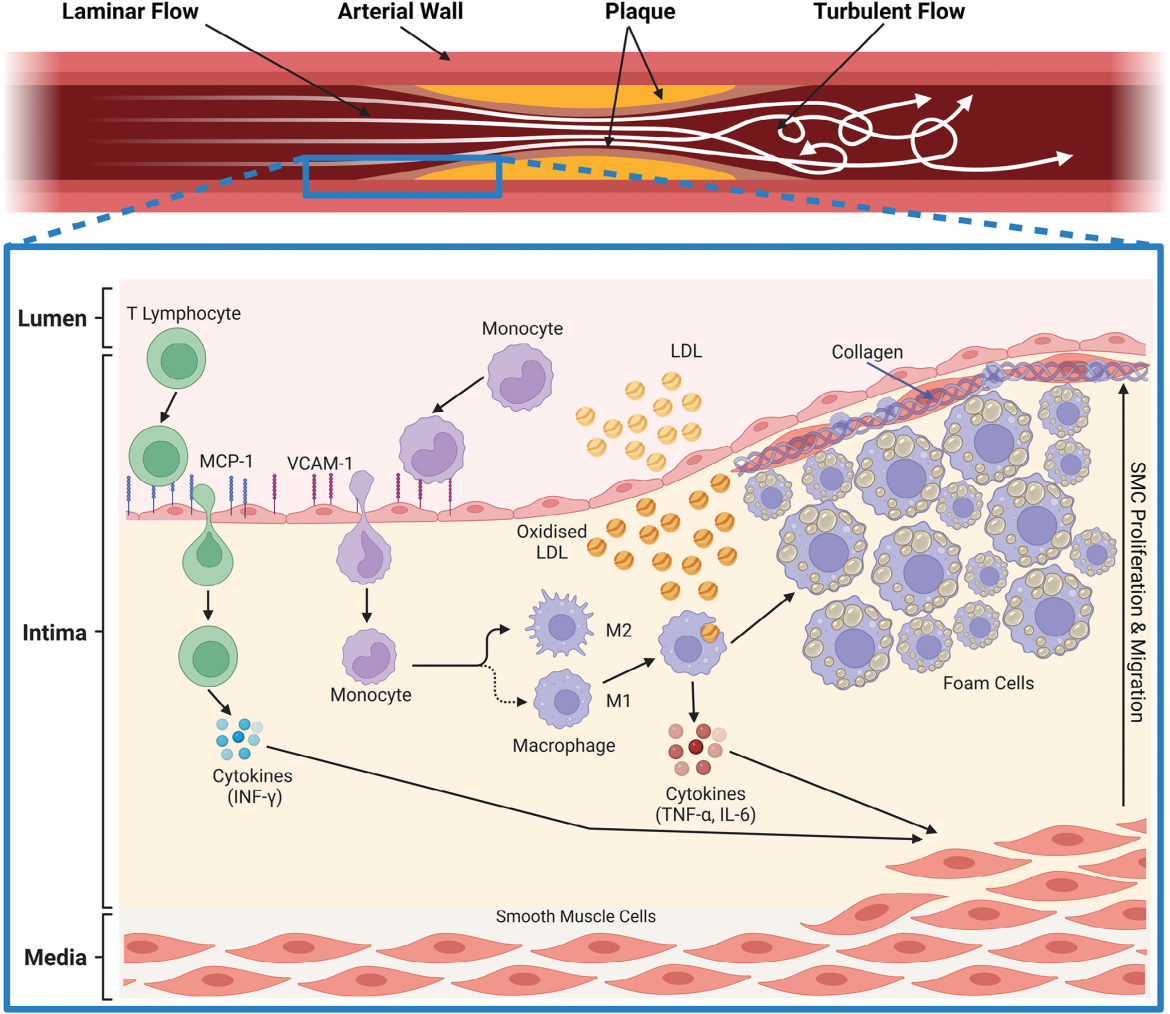
Pathophysiology of Atherosclerosis. Activated endothelium expresses vascular cell adhesion molecule‐1 (VCAM‐1) and intercellular adhesion molecule‐1 (ICAM‐1).^[^ ^4^ ^]^ The production of monocyte chemoattractant protein (MCP‐1) from endothelial cells (ECs) and smooth muscle cells (SMCs) is also seen. MCP‐1 and VCAM‐1 adhere to monocytes and T‐lymphocytes, respectively, migrating them to the tunica intima.^[^ ^5^ ^]^ Monocytes differentiate into macrophages through the absorption of lipids. A scavenger receptor on the macrophage consumes the oxLDL molecules, and macrophages become foam cells, forming the fatty streak seen in atherosclerotic lesions. At this stage, there are no disruptions in the hemodynamics processes within the vessel wall.^[^ ^6^ ^]^ There are two types of macrophages in the atherosclerosis process: M1 and M2 macrophages, dictating pro‐inflammatory and anti‐inflammatory phenotypes, respectively. M1 macrophages secrete cytokines such as IFN‐***γ***, TNF‐***α***, and IL‐6, whereas M2 macrophages produce IL‐10 and tumor growth factor‐beta (TGF‐***β***), which aims to resolve inflammation and deactivate the former macrophage phenotype.^[^ ^7^ ^]^ The M1 cytokines increase the inflammation around the area and activate ECs to attract more monocytes, thus allowing the foam cell to increase in size and thickens the arterial wall.^[^ ^8^ ^]^ Platelet‐derived growth factor (PDGF) produced by macrophages promotes smooth muscle cell migration and proliferation, which increases collagen concentration in the tunica intima.^[^ ^2^ ^]^ The SMCs form the fibrous cap of plaques and stabilize the plaque; however, when in the presence of TNF‐***α***, IL‐6, oxLDL, and foam cells, SMCs will secrete metalloproteinases (MMP). MMP production results in collagen degradation and the fibrous cap weakening.^[^ ^9^ ^]^ Once the pressure within the plaque passes its threshold, plaque ruptures, and a thrombus is formed. Clotting factors and platelets aggregate in the area and impede blood flow going to and from the heart, thus limiting the transport of nutrients to cardiac muscles.^[^ ^10^ ^]^ Created with Biorender.com.

Atherosclerosis is the thickening and hardening of arteries caused by plaque build‐up in the vessel lumen leading to obstruction of blood flow. It is a multifaceted pathophysiological process involving lipid accumulation, inflammation, and fibrosis. Cardiovascular risk‐associated stimuli, including proinflammatory cytokines and low‐density lipoprotein (LDL), instigate the onset of atherosclerosis. This is most prevalent in curved and bifurcated artery geometries where disturbed flow and oscillating shear stresses promote a pro‐inflammatory phenotype in endothelial cells.^[^ ^1^ ^]^ This leads to the activation of the vascular endothelium, prompting it to express adhesive molecules that facilitate the binding of monocytes and lymphocytes (**Figure** **1**). LDLs are directed toward the intima, where monocytes differentiate into macrophages and absorb lipids to form foam cells, resulting in fatty streaks seen in atherosclerotic lesions clinically. Platelet‐derived growth factor (PDGF) produced by macrophages promotes smooth muscle cell migration, proliferation, and collagen deposition in the tunica intima.^[^ ^2^ ^]^ Smooth muscle cells (SMCs) form fibrous caps that stabilize the plaque. However, due to extreme blood velocity and pressures produced by the narrowing of the vessel, the fibrous cap thins out, increasing the risk of plaque ruptures. Plaque rupture causes thrombosis, leading to obstructed blood flow and cardiac ischemia. It is important to note that recent studies have questioned the evidence that high LDL concentrations are the leading cause of endothelial dysfunction, instead suggesting that elevated triglyceride levels are the main cause in combination with low levels of high‐density lipoprotein (HDL).^[^ ^3^ ^]^

## 3 Computational Modeling of Atherosclerosis

Computational modeling is a powerful tool for studying atherosclerosis formation and its impact on the hemodynamic properties of blood vessels. It can reproduce complex vessel and plaque geometries and capture patient‐specific pathogeneses that are difficult to evaluate through theoretical or experimental methods.^[^ ^11^ ^]^ The development and validation of computational models initially relies on establishing appropriate plaque morphology. This parameter is either modeled based on imaging of individual patient plaques or idealized plaque morphologies. Once the plaque geometries are established, the atheromas can be modeled as occlusions, focusing solely on the flow patterns of the blood and the deformation of the vessel wall. Alternatively, atherosclerotic artery modeling can delve into the specific stages of atherosclerotic initiation and development, modeling where individual biological elements are modeled in the system.

### 3.1 Plaque Morphology and Plaque Imaging

Plaque morphology commonly refers to the shape and size of plaque in the vessel lumen, but ideally, it also captures the plaque position, composition, and mechanical properties, which can all impact hemodynamic processes in the affected vessel. Plaque morphology is unique to each individual, making accurate and accessible imaging of patient‐specific plaque morphology critical in studying and modeling atherosclerosis in computational and in vitro studies. This section delves into various imaging techniques for capturing patient‐specific plaque morphology, facilitating its integration into computational simulations. Idealized plaque morphology is also considered, given the scarcity of available patient data.

#### 3.1.1 Imaging Patient‐Specific Plaque Morphology

Plaque can be observed and identified via multiple imaging and analysis techniques, including histological analyses, 2D and 3D image reconstruction from coronary tomography cardiac angiogram (CTCA), intravascular ultrasound (IVUS), optical coherence tomography (OCT), and magnetic resonance imaging (MRI). Each imaging modality has unique advantages and disadvantages; combined, they offer a complete understanding of plaque morphology and composition. While plaque is unique to each individual, plaque morphology is generally classified into four categories based on phase‐contrast computed tomography (CT) imaging, including 1) plaque with a lipid or necrotic core surrounded by fibrous tissue and with possible calcification, 2) calcified plaque, 3) fibrotic plaque without a lipid core or with possible minor calcification, and 4) complex plaque with possible surface defects, hemorrhage, and thrombus.^[^ ^49^ ^]^ **Table** **1** summarizes the analysis of plaque categories via different analysis modalities.

**Table 1:** Morphology of different classes of plaque was obtained using a range of imaging modalities Reproduced (Adapted) with permission.^[^ ^12^ ^]^ Copyright 2021, Frontiers Media. All histological images are stained with hematoxylin and eosin.

		Histological Analysis		2D Reconstruction		3D Reconstruction	
Calcified Plaque		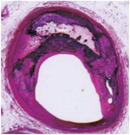	Coronary vessel showing plaque with severe calcification. Reproduced (Adapted) with persmisson.^[^ ^30^ ^]^ Copyright 2007, Japanese Circulation Society	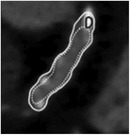	Coronary vessel showing calcified plaque. Image obtained via CTCA. The inner (dotted line) and outer (solid white line) vessel walls and calcified plaque (black line) were identified via binary segmentation techniques. Reproduced (Adapted) with permission.^[^ ^31^ ^]^ Copyright 2018, Elsevier	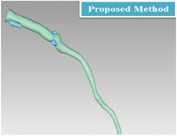	3D reconstruction of a coronary vessel (green) showing calcified plaque (blue) location & morphology. Images obtained via CTCA. Reproduced (Adapted) with permission.^[^ ^31^ ^]^ Copyright 2018, Elsevier
Non‐Calcified Plaque	Lipid or Necrotic Core	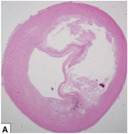	Coronary vessel showing plaque with a large necrotic core covered by a thin fibrous cap. Reproduced (Adapted) with permission.^[^ ^32^ ^]^ Copyright 2018, Elsevier.	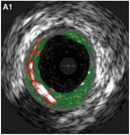	Coronary vessel showing lipid‐core plaque with a necrotic core (red), dense calcification (white), fibrous tissue (dark green), and fibro‐fatty tissue (light green). Image obtained via IVUS. Reproduced (Adapted) with permission.^[^ ^33^ ^]^ Copyright 2017, Lippincott Williams & Wilkins.	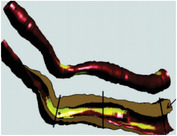	3D reconstruction of the LAD artery showing lipid‐core plaque (yellow). The top is the epicardial vessel surface, and the bottom is a vessel cross‐section showing the endoluminal surface. Images were obtained via multi‐slice computer tomography (MSCT) and IVUS. Reproduced (Adapted) with permission.^[^ ^34^ ^]^ Copyright 2010, Lippincott Williams & Wilkins.
	Fibrotic	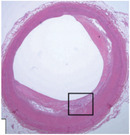	Coronary vessels showing fibrous plaque with large amounts of foam cells on the luminal surface. Reproduced (Adapted) with permission.^[^ ^32^ ^]^ Copyright 2018, Elsevier.	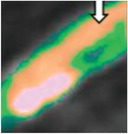	Coronary vessel shows fibrous plaque with a necrotic core (dark green), fibrous plaque (light green), calcified plaque (pink), and the vessel lumen (orange). Image obtained via CTCA. Reproduced (Adapted) with permission.^[^ ^35^ ^]^ Copyright 2017, Elsevier.	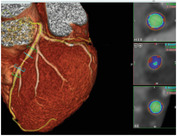	3D reconstruction & semi‐automated plaque analysis in the LAD artery showing plaque containing lipid (red), fibrous tissue (blue), and calcification (yellow). Images on the right are cross‐sections of different artery areas indicated on the left. Images obtained via CTCA. Reproduced (Adapted) with permission.^[^ ^36^ ^]^ Copyright 2017, PLoS.
Complex plaque with surface defects, hemorrhage, and thrombus		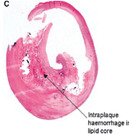	Carotid vessel showing intraplaque hemorrhage in lipid core. Reproduced (Adapted) with permission.^[^ ^37^ ^]^ Copyright 2003, Lippincott Williams & Wilkins.	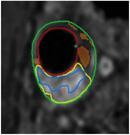	Carotid vessel showing intraplaque hemorrhage (blue) with a necrotic core (yellow), calcifications (brown), outer vessel wall (green), and inner vessel wall (red). Image obtained via MRI. Reproduced (Adapted) with permission.^[^ ^38^ ^]^ Copyright 2019, Lippincott Williams & Wilkins.	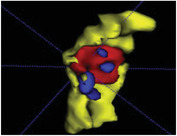	3D reconstruction of plaque within a carotid vessel, showing the plaque (yellow), intraplaque hemorrhage (red), and calcifications (blue). Images obtained via MRI. Reproduced (Adapted) with permission.^[^ ^39^ ^]^ Copyright 2020, Elsevier.

Histology is one of the conventional pathology methods, which uses microscopic analysis of tissue anatomy, and involves sectioning, staining, and imaging patient tissue. It allows the identification of the different plaque components, including individual cell types, inflammation and fibrous tissue formation, the lipid core, and areas of hemorrhage. However, the technique is invasive, requiring an explanation of the patient tissue and sectioning, which can damage plaque resulting in a limited supply of tissue for future studies.^[^ ^13^ ^]^ Importantly, histological analyses only provide a 2D geometry, which does not consider the 3D architecture of the vessels unless further reconstruction is utilized.^[^ ^14^ ^]^ Conversely, plaque can be imaged in vivo using CTCA, IVUS, OCT, or MRI analysis to obtain 2D scans of diseased vessels from multiple angles. These images can be reconstructed using image analysis software to create 2D or 3D representations of the stenosed arteries.

CTCA is a routine, minimally invasive CT scanning technique to diagnose atherosclerosis.^[^ ^15^ ^]^ However, image quality and resolution are limited in small vessels such as the coronary artery, and cardiac motion can introduce imaging artifacts.^[^ ^16^ ^]^ CTCA analysis can distinguish between calcified and non‐calcified plaque but cannot identify and distinguish any subcomponents such as lipid‐rich or fibrotic plaque.^[^ ^17^ ^]^

IVUS consists of a catheter with a miniaturized ultrasound probe deployed to the vessel of interest for imaging and diagnosis. Unlike CTCA, which images the vessel lumen and may under‐ or over‐estimate the boundaries of the plaque, IVUS allows direct imaging of the vessel wall and has a higher resolution than CTCA.^[^ ^18^ ^]^ With emerging technology, virtual histology intravascular ultrasound (VH‐IVUS) can assess tissue composition and classify plaque based on volume percentages of fibrotic tissue, necrotic core, and calcium, while CTCA imaging cannot.^[^ ^19^ ^]^

OCT is the optical analog of IVUS using near‐infrared light absorbed by red blood cells, water, lipids, and proteins at a low level.^[^ ^20^ ^]^ IVUS has a higher tissue penetration and can assess the entire structure of a stenosed coronary artery, such as larger plaques and the vessel wall.^[^ ^21^ ^]^ Still, OCT has a higher resolution, allowing for a more precise assessment of endoluminal structures within coronary arteries.^[^ ^21^ ^]^ OCT imaging can identify different plaque components via their light‐polarizing properties.^[^ ^20^ ^]^ For example, OCT imaging of 183 patients concluded that lipid‐rich plaques with high macrophage numbers had a more significant progression and higher risk of plaque rupture than calcified plaques.^[^ ^22^ ^]^ Additionally, OCT data provides better resolution required for further 3D reconstruction to analyze in computational simulations than IVUS data.^[^ ^21^ ^]^ Other improvements in imaging techniques have integrated IVUS and OCT to create a hybrid system combining IVUS for depth of penetration and OCT for high resolution.^[^ ^23^ ^]^

MRI is a non‐invasive imaging technique that uses magnets and radiofrequency waves to detect areas of soft tissue to obtain detailed anatomical images.^[^ ^24^ ^]^ MRI is more sensitive in detecting a necrotic core and hemorrhage than other modalities in carotid arteries ^[^ ^25^ ^]^ and has superior soft tissue contrast and spatial resolution, offering substantially enhanced images of stented iliac arteries compared with IVUS and OCT.^[^ ^26^ ^]^ Intravascular MRI allowed imaging of distinct diseased human coronary arteries showing thickened vessel walls and plaque presence, but further imaging resolution improvements are required to segregate individual plaque components.^[^ ^27^ ^]^ Intravascular MRI requires longer scan times than other modalities, especially when extensive coverage of the vessel is needed,^[^ ^28^ ^]^ and respiration from patients creates motion artifacts that hinder the resolution of images, limiting the acquisition window.^[^ ^29^ ^]^

Data from these imaging techniques can be reconstructed into information about the plaque location, shape, and composition and used to develop patient‐specific computational models of atherosclerotic vessel obstructions.

#### 3.1.2 Idealized Plaque Morphology

**Figure 2:**
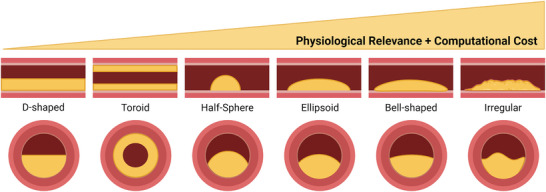
Idealized plaque morphologies. More physiologically relevant morphologies also carry a higher cost in computational power, time, and memory required to model. Created with Biorender.com.

Due to the scarcity of patient‐specific plaque morphology data and the resource‐intensive nature of individual patient imaging and image reconstruction, many researchers have opted to use idealized versions of the shape and location of plaque within the coronary vessel (**Figure** **2**) when developing computational models of atherosclerosis. Idealized geometries simplify plaque morphology to D‐shaped (semi‐spherical), concentric (homogenous obstruction around the vessel wall with the center line matched to the vessel), and eccentric (homogenous obstruction around the vessel wall with the center line mismatched to the vessel) shapes at different degrees of stenosis.^[^ ^40^ ^]^ These simplified geometries allow researchers to study the effect of plaque morphology and size on blood flow. For example, concentric circular stenosis was shown to have fewer hemodynamic complications compared to more complex shapes such as eccentric half‐moon morphology. However, concentric morphologies are less typical than their eccentric counterparts, as demonstrated by 73% of coronary arteries analyzed in one study having eccentric plaque.^[^ ^41^ ^]^ Further modeling of coronary arteries revealed higher wall shear stress (WSS) at the center line of a longitudinal view seen in eccentric plaques compared to concentric plaques.^[^ ^42^ ^]^ Higher WSS has been previously associated with high plaque vulnerability and progression of lipid core growth.^[^ ^43^ ^]^

To provide a more morphologically accurate model of plaque, different shapes, such as a half‐sphere,^[^ ^44^ ^]^ ellipsoid,^[^ ^65^ ^]^ or Gaussian functions ^[^ ^46^ ^]^ have also been utilized (Figure 2). A review looking at the biomechanics of idealized plaque morphology can be found in.^[^ ^47^ ^]^ Although these idealized geometries allow for preliminary analysis of how differing plaque morphology affects Newtonian and non‐Newtonian flow models within coronary vessels, they still lack the irregular morphology of natural plaque and curvature and branching of a normal coronary artery. They thus cannot accurately replicate the hemodynamics of the system.^[^ ^48^ ^]^ To address this, irregularity of plaque morphology was introduced by creating axially non‐symmetrical but radially symmetrical stenosis, demonstrating the asymmetric flow of blood through a catheterized artery.^[^ ^49^ ^]^ However, this study did not model comparisons with simple plaque shapes and eccentricities. Areas of bifurcation and curvature in the vessel wall exhibit hemodynamic alternations, making these areas more susceptible to atherosclerosis; therefore, in another study, the plaque was modeled on a bifurcated coronary artery.^[^ ^50^, ^51^ ^]^ Bifurcated vessels exhibited areas of low WSS followed by high circulation zones, which is the ideal environment to promote plaque growth. As a result, most atherosclerotic lesions are found near bifurcations or curvatures of arteries.^[^ ^52^ ^]^ A more detailed review of the effects of bifurcations, trifurcations, and curvature in coronary stenosed and healthy arteries can be found in.^[^ ^47^, ^53^ ^]^ The morphology of plaques has been improved where artificial stenoses are constructed using computational fluid dynamics (CFD). The irregular, 3D stenoses are created using previous knowledge of WSS exhibited in healthy arteries.^[^ ^50^ ^]^ A comparison of simple‐shaped plaque geometries using ellipsoids and irregular stenoses demonstrated the irregular, 3D stenoses to have more physiologically relevant morphology and location based on WSS values.

### 3.2 Modeling Hemodynamics in Occluded Coronary Arteries

This section details computational simulations exclusively focused on stenosed coronary arteries in blood flow settings without incorporating any biological agents. Accurately modeling the hemodynamics of stenosed coronary arteries is important to better understand the initiation and progression of atherosclerosis.

Two critical hemodynamic parameters affecting atherosclerosis are WSS and cyclic circumferential strain.^[^ ^54^ ^]^ WSS refers to the tangential force along the wall of the tunica intima.^[^ ^55^ ^]^ Previous research into plaque formation and growth has identified low and high WSS observed on the vessel wall and plaque as critical indicators of atherogenesis and plaque rupture, respectively.^[^ ^56^, ^57^, ^58^, ^59^, ^60^, ^61^ ^]^ Low WSS or stagnation regions are associated with high LDL accumulation and sites of plaque growth,^[^ ^62^ ^]^ whereas high WSS is associated with high plaque vulnerability and increased chance of plaque rupture,^[^ ^63^, ^64^ ^]^ Based on current literature, WSS is considered low at <1.0 Pa and high at >2.5 Pa, but it is important to note that these absolute values may be inaccurate and relative values based on patient data should be considered.^[^ ^60^ ^]^ Cyclic circumferential strain refers to the repetitive pulsatile forces that act upon the vessel wall, which has been shown to enhance plaque formation by stimulating more atheromas to adhere to atherogenic blood vessels.^[^ ^54^ ^]^ Several parameters, including time‐averaged wall shear stress (TAWSS), oscillatory shear index (OSI), and fractional flow reserve (FFR) are significant in atherosclerotic hemodynamic studies. TAWSS signifies the mean wall shear stress, while OSI assesses variations in shear stress to identify regions of high and low flow recirculation, both measured over one cardiac cycle.^[^ ^65^ ^]^ Generally, areas characterized by low WSS exhibit elevated OSI values.^[^ ^66^ ^]^ The FFR holds clinical importance as it represents the ratio between proximal and distal pressures within an artery, serving as a diagnostic tool to ascertain the severity of stenosis.^[^ ^67^ ^]^ A severe stenosis is indicated with an FFR value over 0.8, necessitating intervention through surgery or other alternative treatments. Studies have also indicated that low endothelial shear stress values and helical flow patterns may also be determinants of coronary plaque progression and vulnerability.^[^ ^68^, ^69^, ^70^, ^71^ ^]^

**Figure 3:**
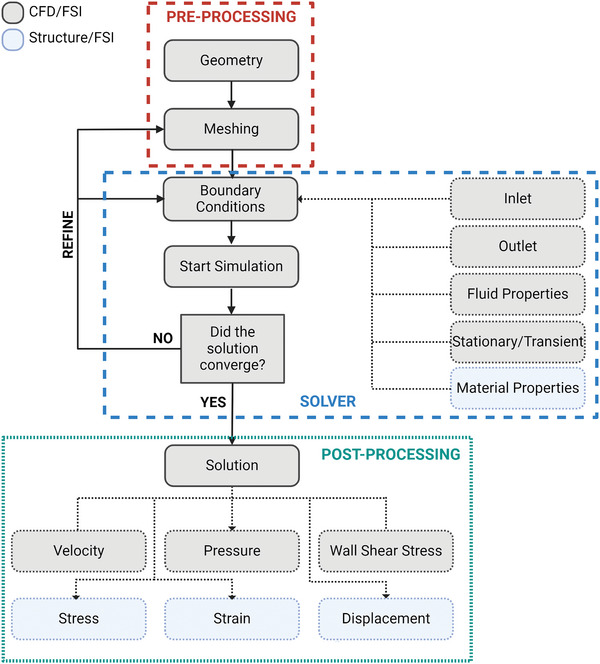
Flow chart illustrating the methodology of computational fluid dynamics (CFD) and fluid‐structure interactions (FSI) simulations. The process involves three distinct stages: 1) preprocessing, 2) solver, and 3) post‐processing. Pre‐processing involves the utilization of computer‐aided design (CAD) software for replicating the geometry of the arterial wall and plaque followed by the generation of a mesh to define the computational domain. The solver involves specifying relevant boundary conditions to simulate physiological fluid behavior and material properties in coronary arteries and the input of boundary and solver conditions to establish the simulation environment. At the completion of the simulation, convergence is required to be achieved to ensure reliable results. Convergence is the point in a simulation where the iterative solution stabilizes and have consistent and unyielding results. If convergence is not attained, refinement in meshing or adjustment of boundary conditions is needed. The results are then extracted from the simulation to analyze changes in velocity, pressure, and wall shear stress waveforms. The examination of stress, strain, and displacement of the arterial wall can be achieved in FSI simulations. Created with Biorender.com.

Two simulation techniques can be used to study the hemodynamics within coronary arteries: computational fluid dynamics (CFD) and fluid‐structure interactions (FSI) modeling. CFD modeling focuses solely on the fluid domain and performs rigid body simulations, ignoring the viscoelastic behavior of the vessel wall.^[^ ^72^ ^]^ FSI modeling couples fluids and structures in a multiphysics simulation that considers blood flow and vessel wall mechanics,^[^ ^62^ ^]^ which are known to affect blood flow in atherosclerosis in coronary arteries.^[^ ^73^ ^]^ The methodology to produce these simulations is similar for both types of modeling (**Figure** **3**).

Atherosclerotic arteries are modeled by adding an obstruction within a healthy artery in CFD and FSI models or by reconstructing images of patient‐specific stenosed coronary arteries (**Table** **2**). To complete these simulations, boundary conditions are vital in formulating a replica of the human condition. These boundary conditions include the flow type, fluid type, wall conditions, and inlet and outlet conditions. Boundary conditions have been identified as critical to computational simulations as their accurate specification is pivotal for ensuring the reliability and validity of simulations.^[^ ^65^, ^74^, ^75^ ^]^ Ongoing discourse within the research community revolves around the standardization of boundary conditions and modeling protocols.^[^ ^76^ ^]^ Nevertheless, researchers are advised to perform individual verification, validation, and uncertainty quantification (VVUQ) studies to enhance the robustness and credibility of their simulations.

**Table 2:** Comparison of Boundary conditions and Validation Methods using CFD/FSI Models of Atherosclerosis. Adapted with permission.^[^ ^47^ ^]^

Geometry	Software	Type of Flow	Fluid	Wall Conditions	Inlet Conditions	Outlet Conditions	Validation	Reference
Patient‐Specific (3D)	MATLAB and ADINA	Laminar	Newtonian	Anisotropic and Isotropic Materials with Cyclic Bending	Physiological pressure conditions	Physiological pressure conditions	None	[78]
Patient‐Specific (2D and 3D)	ANSYS CFX	Laminar and turbulent (Wilcox k‐ω model)	Newtonian	Rigid	Transient velocity waveform	Standard pressure	None	[115]
Patient‐Specific (3D)	CFD‐GEOM and CFD‐ACE	Laminar	Newtonian	Rigid	Velocity waveform from Doppler probe	Pressure waveform from Doppler probe	None	[79]
Patient‐Specific (3D)	Blender and ANSYS CFX	Laminar	Newtonian	Rigid	Pulsatile Velocity waveform using Fourier series	Zero pressure gradient	None	[83]
Patient‐Specific (3D)	Blender and ANSYS CFX	Laminar	Newtonian	Rigid	Transient flow waveform	Zero pressure gradient	None	[84]
Patient‐Specific (2D)	MATLAB	Laminar	Newtonian	Rigid	Velocity waveform from ultrasound Doppler probe	Zero pressure gradient	None	[14]
Patient‐Specific (3D)	ADINA	Laminar	Newtonian	Anisotropic with Cyclic Bending	Velocity waveform from Doppler probe	Pressure waveform from Doppler probe	None	[98]
Patient‐Specific (3D)	ADINA	Laminar	Newtonian	Anisotropic with Cyclic Bending	–	–	None	[94]
Idealized (3D)	ANSYS CFX	Laminar	Newtonian	Increased stiffness of vessel wall	Time‐varying velocity waveform from IVUS Doppler probe	Time‐varying pressure waveform from IVUS pressure probe	Validated by catheter measurement of proximal and distal parts of blood vessel	[95]
Patient‐Specific (3D)	MIMICS and COMSOL	Laminar	Newtonian	Rigid	Mean flow rate	Total Resistance	Fraction flow reserve (FFR) measured by thermodilution of 3D‐printed phantom was compared against CFD‐derived FFR.	[108]
Patient‐specific (3D)	MIMICS and ANSYS CFX	Turbulent (SST k‐ω model)	Non‐Newtonian (Bird‐Carreau Model)	Rigid	Transient pulsatile velocity waveform	Static pressure boundary of 80 mmHg	None	[90]
Patient‐Specific and Idealized (3D)	MIMICS and ANSYS CFX	Laminar	Newtonian and non‐Newtonian (Carreau model)	Rigid	Steady velocity waveform and pulsatile velocity waveform	Based on flow partition in in vitro experiments	Results from the simulation were compared against in vitro experimentation with the 3D‐printed phantom.	[110]
Idealized (3D)	ANSYS FLUENT	Laminar and turbulent (SST k‐ω model)	Newtonian and non‐Newtonian (Carreau model)	Rigid	Constant inlet velocity and instantaneous mean velocity of the cardiac cycle	Zero‐gauge pressure condition	Results from the simulation were compared against 3D printed phantom that was connected to a microscope	[111]
Idealized (3D)	ANSYS FLUENT	Laminar	Non‐Newtonian (Carreau model)	Young's modulus of 3.77 MPa, the density of 1120 kg/m^3^ and Poisson's ratio of 0.49	Physiologically accurate pulsatile velocity profile	Pressure of 80 mmHg	None	[72]

The flow type within CFD and FSI simulations can be either laminar or turbulent. Laminar flow conditions typically describe a smooth and streamlined flow pattern characterized by a low Reynolds number. In contrast, turbulent flows are more irregular and erratic and are defined by a high Reynolds number.^[^ ^77^ ^]^ The flow type within simulations also depends on the inlet and outlet conditions. Inlet conditions are more critical as they define the blood flow being pumped into the geometry and can be either steady‐state or pulsatile flow. Earlier studies have typically relied either on laminar and pulsatile flow conditions or turbulent and steady‐state flow conditions due to the high computational cost of combining turbulent and pulsatile flow models.^[^ ^78^, ^79^ ^]^ However, studies have demonstrated the transition from laminar to turbulent flow in stenosed arteries with over 50% obstruction. During peak systole, a significant difference of 304.6 Pa maximum WSS was observed in a 70% stenosed artery model when comparing the laminar against the turbulent model.^[^ ^80^ ^]^ Previously, steady‐state flow conditions were utilized for atherosclerotic vessel simulations; however, they severely underestimated the pressure, velocity, and WSS in stenosed vessels.^[^ ^81^ ^]^ In vessels with greater than 50% stenosis, low‐velocity fluctuations (0–2.18 mm s^−1^) were shown in post‐stenotic regions, leading to deformation and movement of the coronary wall.^[^ ^82^, ^83^ ^]^ Further, high velocity and WSS (greater than 2.5 Pa) in arteries with greater than 70% stenosis were also detected.^[^ ^84^ ^]^ However, these simulations consisted of Newtonian fluid and do not accurately represent coronary artery blood flow.

The use of Newtonian versus non‐Newtonian fluid in CFD and FSI simulations has been a long‐standing debate in the field.^[^ ^47^ ^]^ Although blood has been defined as non‐Newtonian and should be considered in simulations,^[^ ^85^ ^]^ the high computational cost is a challenge to overcome. Studies have shown that while Newtonian fluids are used in simulations of larger arteries, non‐Newtonian fluids better represent blood flow in smaller artery simulations, especially those with high stenosis severity.^[^ ^86^, ^87^ ^]^ Non‐Newtonian fluid accounts for the changes in viscosity that are lacking in a Newtonian fluid and, therefore, more accurately estimates WSS in disturbed flow regions.^[^ ^88^, ^89^ ^]^ One study found that non‐Newtonian turbulent fluid in blood vessels with stenosis severity over 40% showed high WSS at the peak of the stenosis, which may lead to plaque rupture.^[^ ^90^ ^]^ Conversely, areas of low shear stress contribute to the formation of intimal lesions.^[^ ^91^ ^]^ Similarly, analysis of histological IVUS images of diseased arteries demonstrated that low WSS regions developed larger plaque and necrotic core progression. In contrast, high WSS regions (4.39 ± 2.14 Pa) show fibrous and fibro‐fatty tissue regression and excessive expansive remodeling.^[^ ^79^ ^]^ Different models of non‐Newtonian flow have also been studied in reconstructed stenosed and non‐stenosed LAD models, demonstrating Carreau, modified Casson, and Quemada blood models were the most appropriate to calculate blood viscosity.^[^ ^92^ ^]^ An alternative study questions whether the investigation into the effects of fluid shear viscosity merits consideration after findings of no discernible distinctions in WSS and helical flow values during the exploration of Newtonian and non‐Newtonian flow in 288 CFD simulations of reconstructed angiogram geometries.^[^ ^93^ ^]^

Most CFD simulations assume vessel wall rigidity and do not consider cyclic circumferential strain or cyclic bending. Ignoring the vessel wall's cyclic movement means that the complete dynamics of blood cannot be captured, which prevents deeper insight into the blood flow and plaque interaction. Therefore, FSI modeling should be used despite requiring more computational power. A 3D patient‐specific model developed from IVUS imaging was imported into FSI software with a simplified pressure profile and periodic displacements of the lower edge of the vessel. The model demonstrated a decrease in the maximum shear stress (15%), maximum flow velocity (5%), and flow rate (8.7%) within the system despite the lack of recirculation, which is not observed in simple CFD simulations.^[^ ^78^, ^94^ ^]^ Although the FSI simulations were developed using patient‐specific images of stenosed arteries, the blood vessel boundary conditions and material properties were all assumed or populated with values from the literature. Therefore, further experimentation is required using accurate fluid profiles and vessel wall approximations.

To improve the accuracy of simulations, an FSI model of a curved coronary vessel with different levels of stenosis (0% to 90%) incorporated vessel stiffness proportionate to the stenosis severity.^[^ ^95^ ^]^ As stenosis increased, there was a positive correlation between local WSS (60%, 70%, 80%, and 90% stenosed is 1.41, 1.71, 2.41, and 5.06 times higher than healthy values) and wall pressure (60%, 70%, 80%, and 90% is 1.54, 2.11, 3.44, 7.33, and 30.41 times higher than healthy values), which increases the blood flow resistance and the vulnerability of plaque at the site of stenosis. However, it is also essential to consider how local wall thickness changes the mechanical properties of the wall, as well as incorporating factors such as cyclic bending and the elastic properties of the vessel wall.^[^ ^96^ ^]^ Previous studies have shown that using anisotropic materials using non‐linear material models for the vessel wall can result in a 400% increase in stress production.^[^ ^97^ ^]^ Therefore, using patient‐specific coronary vessels constructed from MRI images with anisotropic vessel materials and incorporating cyclic bending and pulsatile blood flow can lead to more physiologically accurate boundary conditions.^[^ ^98^ ^]^ However, the majority of the coronary vessels are simulated as hyperelastic isotropic material using a reduced Polynomial model or Mooney–Rivlin model due to limitations in computational cost.^[^ ^99^ ^]^ Although high shear stress caused a strain increase in human coronary plaques,^[^ ^81^ ^]^ in vivo stress conditions, plaque component material properties, on‐site pressure, flow information, and data at the cellular and molecular levels were not incorporated. Additionally, the number of patients required to validate these experiments would result in high costs and immense difficulties in patient recruitment.^[^ ^100^ ^]^ Conversely, studies have ascertained that wall elasticity and cardiac motion may be excluded in modeling studies, as shown by no significant differences in TAWSS values, with only marginal disparities in the temporal variation of WSS.^[^ ^101^, ^102^ ^]^ This prompts a discourse on the consideration of whether augmenting modeling parameters with additional complexities contributes to an enhanced accuracy of the model itself.

Several studies have considered the mechanical properties of plaque in addition to the dynamics of the vessel wall. One study used calcified and non‐calcified idealized plane models of atherosclerosis. The plaques were modeled as non‐homogenous and anisotropic, with distinct fibrous tissue, lipid core, and calcium areas, each assigned Young's modulus and Poisson's ratio. An FSI model was then created to analyze the plaque and vessel wall dynamics under different clinical conditions.^[^ ^103^ ^]^ The model shows the plaque's peak deformation and critical stress increases as the fibrous cap thins in non‐calcified carotid plaques. With a fibrous cap thickness of 0.05 mm and lipid core of 0.35 mm, the maximum deformation of the plaque measured 0.467 mm, whereas a fibrous cap thickness of 0.48 mm yielded a lower deformation at 0.0281 mm. Other experiments viewed each plaque component as a rigid body with one mechanical force acting on the body and extrapolated Young's modulus and Poisson's ratio from previous literature.^[^ ^104^ ^]^ It was determined that maximal stress and stress concentrations around the vessel wall were significantly higher in ruptured lesions than in non‐ruptured ones. Comparatively, different mechanical properties values were observed in data obtained directly from patients.^[^ ^105^ ^]^ Different non‐linear models such as neo‐Hookean, Mooney Rivlin, Holzapfel–Gasser–Odgen, Polynomial, Arruda–Boyce, Yeoh, and Gent can be used to simulate the deformation nature of the plaque. The choice of the model to represent plaque is dependent on the optimization of coefficients in the model.^[^ ^106^ ^]^ Therefore, to eliminate the uncertainty in using numerical mechanical properties values, lipid and calcium components were modeled as incompressible Neo‐Hookean solids. The fibrous intima and arterial wall were modeled as incompressible Yeoh materials to capture the non‐linear mechanical behavior expressed in natural arteries and were compared to previous mechanical characterizations of coronary arteries, which yielded similar results.^[^ ^107^ ^]^ However, it is essential to note that FSI models have various assumptions in boundary conditions and mesh size and shape. These studies were not validated against previous literature or through further experiments.

The limited availability of clinical data is a significant concern for computational simulation validation. To overcome this challenge, many researchers have used mechanical models that utilize 3D‐printed phantoms to mimic the morphology of diseased vessels. The phantoms are developed from different materials to capture vessel and plaque properties, and the hemodynamics are studied by connecting the phantom to a circulation loop. A study using simplified geometric models of diseased coronary arteries from 8 patients demonstrated a good correlation between 3D‐printed and 3D‐computational models and observed that low‐severity stenoses were typically found in locations further away from the point of bifurcation.^[^ ^108^ ^]^ 3D printed resin models also allow quantification of spatial characteristics of the stenosis, such as the severity of stenosis, the exact locations of the lesions, the angle of bifurcation, and the curvature of the bifurcation. However, it should be noted that the small sample size of the experiments and the rigid and brittle nature of the material used to print the 3D models limit their utility in studying vessel hemodynamics.

Idealized geometries are currently much more accessible to print than patient‐specific geometries^[^ ^109^ ^]^; however, irregular geometry and material properties of the vessel wall and plaque are required to study the natural morphology of plaque and blood vessels. CFD simulations of artificially stenosed idealized and patient‐specific models were shown to have an increase in flow velocity around the area of stenosis, which is more aligned with velocities observed in natural coronary arteries.^[^ ^110^ ^]^ 3D‐printed polydimethylsiloxane (PDMS) models of the CFD geometry were produced, and through epifluorescence microscopy, micro particle image velocimetry measurements were obtained and found to be similar to the CFD simulations. Additionally, a study utilized 3D‐printed resin models with varying degrees of stenosis and non‐linear material properties to represent the vessel wall more accurately than a rigid material. Changes in WSS and fluid velocity were observed and compared to FEA models, which proved to be in close agreement with one another.^[^ ^111^ ^]^ Despite the ability of 3D‐printed phantoms to mimic the morphology of diseased and healthy vessels, the compliance and shear stress calculated may differ depending on the materials utilized to print the vessel. This approach is also limited by time constraints and the need to manually remove imperfections.^[^ ^112^ ^]^

Computer simulations have greatly aided in understanding the development and progression of atherosclerosis. These simulations have allowed researchers to examine blood flow and fluid mechanics through differing stenosed vessels and have shown the effects of vessel wall compliance, plaque mechanics, and irregular plaque morphology on the flow patterns in coronary arteries. The use of 3D‐printed rigid models allows for changes in WSS and fluid velocity to be observed in vitro, providing a helpful tool for spatial characteristic quantification and validation. Although computer simulations allow for the controlled and systemic study of atherosclerosis, they have limitations, including the difficulty of incorporating material behavior changes, using approximations and assumptions during simulations, and the high computational cost for complex geometries.^[^ ^113^ ^]^ To address the limitations of computer simulations, 3D bioprinting, which involves creating coronary arteries exhibiting native physiochemical and biomedical characteristics, is becoming increasingly utilized.^[^ ^114^ ^]^

### 3.3 Modeling Hemodynamics during Atherosclerotic Initiation and Development

This section outlines atherosclerotic models focusing on incorporating biological molecules as mathematical equations to look at plaque formation through LDL accumulation, plaque growth, and plaque rupture. Most mathematical models start with diffusion and convection equations of atherosclerotic evolution, from steady state flow using laminar fluid, then adding different biological elements to the equation and including a time‐dependent variable to control the growth over a period of time.^[^ ^116^ ^]^ There has been research into alternative models, including the integration of agent‐based models (AGM) with CFD models to simulate arterial wall remodeling and replicate physiological and pathological features in atherosclerotic arteries for more accurate models of plaque growth.^[^ ^117^, ^118^, ^119^ ^]^ However, with agent‐based models, a compromise between the resolution of each biological element and computational cost needs to be conducted initially.^[^ ^120^ ^]^

Initially, boundary conditions are first established, followed by identifying the number of layers and interfaces used in the model, the type of fluid used (Newtonian or non‐Newtonian), the type of flow (laminar or turbulent), and inlet and outlet boundary conditions.^[^ ^121^ ^]^ There has been discourse between researchers about using Newtonian and non‐Newtonian flow within simulations as mentioned in Section 3.2. Some agree that Newtonian fluid is a reasonable assumption when simulating blood flow in obstructed arteries with some care taken.^[^ ^122^ ^]^ However, others believe that due to the turbulent nature of the flow, non‐Newtonian fluid is required to identify micropatterns of flow disturbances in stenosed arteries and is more representative of natural human physiology.^[^ ^89^ ^]^

Different processes within the atherogenesis inflammatory response can be modeled, such as LDL oxidation,^[^ ^123^ ^]^ monocyte chemoattractant protein‐1 secretion,^[^ ^124^ ^]^ monocyte recruitment,^[^ ^125^ ^]^ monocyte to macrophage differentiation,^[^ ^126^ ^]^ foam cell formation and accumulation,^[^ ^127^ ^]^ T‐cell recruitment and the role of interferon‐γ,^[^ ^127^ ^]^ the proliferation of SMCs,^[^ ^125^ ^]^ and collagen formation.^[^ ^128^ ^]^ Ordinary differential equations or partial differential equations of these processes are typically used to simulate plaque growth and can be used to predict plaque rupture events using 2D^[^ ^129^ ^]^ or 3D geometry.^[^ ^130^ ^]^ To demonstrate this, a study utilized one‐way FSI to model atherosclerotic growth, which incorporated vessel wall thickening, lipoprotein transport through the arterial wall, and inflammation modeling via ordinary differential equations and diffusion and convection equations to evaluate endothelial shear stress throughout the geometry.^[^ ^131^ ^]^ This study assumed that shear stress does not change with wall thickening; however, a study involving 21 male subjects demonstrated an inversely proportional relationship between shear stress and intima‐media thickness in carotid arteries.^[^ ^132^ ^]^ Further, due to the simplicity of the simulation, features such as depressions or peaks of WSS, especially those associated with the coronary flow where there is a period of almost negligible flow, would not be seen in this model. Further improvements in developing a two‐way FSI model would be recommended, as the interface between the lumen and adventitia can also be analyzed. A more detailed review of the mathematical models of atherosclerotic growth can be found in refs. [121] and [133].

Literature has explored LDL transport models in detail, aiming to understand factors influencing LDL accumulation and their potential as predictors for atherosclerotic disease progression,^[^ ^134^, ^135^ ^]^ LDL transport models typically fall into two categories: lumen or wall‐free models, where the particles within the fluid domain are considered only, and fluid‐wall models, where both the lumen and arterial wall are simulated. In one study, a fluid‐wall model showed high LDL concentrations were identified distal to the plaque locations, aligning with clinical observations.^[^ ^62^, ^136^ ^]^ This study also observed the impact of hypertension, correlating with an increased prevalence of high LDL regions. Another investigation employed a three‐pore fluid‐wall model simulating plasma flow and LDL transport through the endothelial layer within the arterial wall.^[^ ^137^ ^]^ Although high levels of LDL accumulated at disturbed flow areas were identified, high computational cost was also incurred. Another innovative study departed from conventional advection‐diffusion equations for LDL transport and instead utilized WSS quantities to predict mechanistic links between WSS and LDL transfer at the arterial blood–wall interface, while concurrently reducing the computational cost.^[^ ^138^ ^]^ The prior studies operated under steady‐state flow conditions; however, a separate study demonstrated the significance of pulsatile flow, noting that steady‐state flow might underestimate LDL and albumin accumulation areas.^[^ ^139^ ^]^ Investigations into atherosclerotic side branches of coronary arteries have also been conducted, revealing elevated LDL concentrations in coronary arteries with stenotic side branches.^[^ ^140^ ^]^ This heightened concentration increases the risk of intervention due to a greater likelihood of plaque growth and rupture. Higher order models, such as biochemical transport models, incorporating mass transport of different molecules involved in atherosclerosis and the incorporation of lipidemics present promising outlooks into combining different sets of data to predict site‐specific progression of coronary atheromas.^[^ ^141^, ^142^ ^]^

Therefore, models based on biological processes allow for natural shapes of stenosis and multiple parameters to be included, such as arterial vessel wall permeability, diffusion constants, etc., thus providing more complexity to the model and more accurate hemodynamics of blood flow can be observed.^[^ ^50^ ^]^ However, due to atherosclerosis being a very complex biological process, many agents and substances are neglected due to a lack of information in the current literature.^[^ ^143^ ^]^ Furthermore, more research into different geometries, with bifurcations and patient‐specific morphologies, and incorporating more dynamics to the arterial wall movement, is required to improve the accuracy of mathematical models.^[^ ^143^ ^]^ Validation of the mathematical models is lacking in many articles, with some providing some validation with previous literature; however, in vitro experimentation and/or comparisons with clinical data would be preferable.^[^ ^144^ ^]^

## 4 Biological Modeling of Atherosclerosis

**Figure 4:**
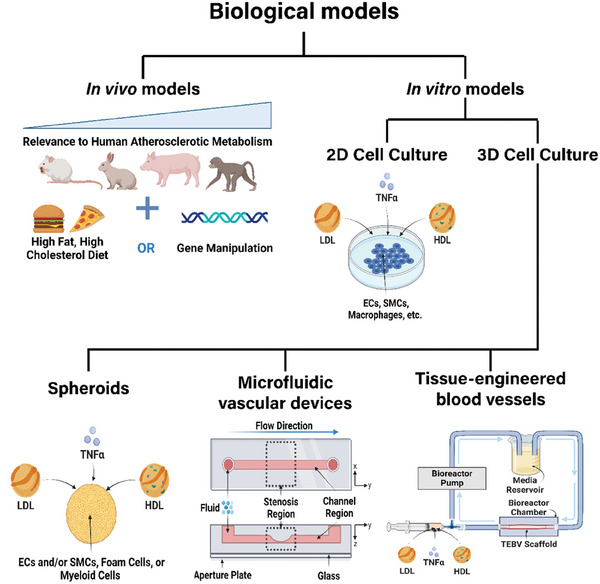
Overview of different types of biological models of atherosclerosis. The biological models for atherosclerosis can be broadly classified into in vivo (living animal models) and in vitro (laboratory‐based models). Atherosclerosis is primarily induced in animals such as mice, rabbits, pigs, and non‐primate models by prolonged consumption of a high‐fat, high‐cholesterol (HFHC) diet, or by initial gene manipulation followed by an HFHC diet. In vitro models, such as 2D and 3D cell cultures, are used to explore the molecular and cellular mechanisms of atherosclerosis. In 2D cell cultures, cells like endothelial cells (ECs), smooth muscle cells (SMCs), and macrophages are grown on a flat surface and exposed to various factors like low‐density and high‐density lipids (LDLs and HDLs) and tumor necrosis factor‐alpha (TNF‐α), which mimics atherosclerotic changes such as endothelial dysfunction, foam cell formation, and plaque development. Conversely, 3D cell culture models are more advanced, offering a better representation of physiological conditions in the arterial wall. These models can be divided into spheroids, microfluidic vascular devices, and tissue‐engineered blood vessels. Atherosclerotic factors such as LDLs, HDLs, and TNF‐α are added to the system to induce atherosclerosis. Due to their added structure, 3D models offer a more accurate representation of hemodynamic factors like shear stress and fluid flow, cell‐to‐cell interactions, and cell signaling pathways involved in atherosclerosis. Created with Biorender.com.

Biological models are constructed to gain an in‐depth understanding of human physiology or disease. Biological models can be in vivo (living animal models), ex vivo (human or animal tissue maintained outside the normal physiological context, i.e., outside the body), or in vitro (outside the body; simplified laboratory‐based models), describing the testing environment in which they are conducted. The study of atherosclerosis is typically performed in in vivo animal or in vitro 2D and 3D cell culture models (**Figure** 4), as harvesting and maintaining human/animal arteries for ex vivo models in a reproducible manner is technically and logistically challenging.

### 4.1 In Vivo Models

The most common biological models of atherosclerosis involve in vivo studies where atherosclerosis is induced in animals via a cholesterol‐rich diet, genetic manipulation, or by introducing risk factors such as diabetes in the animal.^[^ ^145^ ^]^ Animals are injected with components that induce endothelial dysfunction, such as LDLs, tumor necrosis factor‐alpha (TNF‐α), or HDLs and then monitored over a certain period to demonstrate atherosclerotic growth. Studies of animals with atherosclerosis are also conducted to better understand the impact of hemodynamics, mechanical forces, and other biological factors on the growth and rupture of plaques. These models have provided critical insights into atherosclerosis risk factors, disease initiation and progression, and therapeutic interventions. However, they are resource intensive and are not entirely representative of human disease due to genetic, anatomical, metabolic, and disease progression differences.^[^ ^146^ ^]^

**Figure 5:**
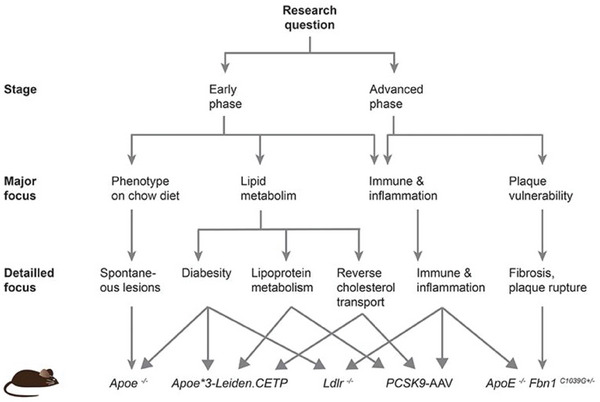
Mouse models of different aspects of atherosclerosis. Reproduced (Adapted) with permission.^[^ ^147^ ^]^ Copyright 2019, Frontiers Media.

Mice are the most common species used in atherosclerosis modeling due to their high reproduction rates, ease of genetic manipulation, and ease of monitoring in a reasonable time frame. Common mouse models of atherosclerosis include 1) Apolipoprotein E deficient (ApoE^−/−^) mice, 2) LDL‐receptor deficient mice, 3) Apolipoprotein E3‐Leiden mice, 4) PCKS9‐AAV mice, and 5) Apolipoprotein deficient mice (ApoE^−/−^) crossbred with mutant fibrillin‐1 allele (Fbn1^C1039G+/−^),^[^ ^145^, ^147^ ^]^ each representing a different stage or aspect of atherosclerosis (**Figure** 5). An in‐depth review of these models can be found in ref. [147].

The initial atherosclerotic mouse models focused on atherogenesis,^[^ ^145^ ^]^ seen typically in ApoE^−/−^ mouse models as they are used to study endothelial dysfunction due to their ability to develop atherosclerotic lesions spontaneously. These models have demonstrated that endothelial dysfunction is influenced by sex, age, and the type of diet consumed.^[^ ^148^ ^]^ LDLr^−/−^ and ApoE*3‐Leiden.CETP and PCSK9‐AAV models can be used to study lipid metabolism and inflammation stages of atherogenesis. LDLr^−/−^ models exhibit fatty streak plaques that turn into fibrous plaques with an accumulation of macrophages, as usually seen in the early stages of atherosclerosis and similar to what is observed in humans.^[^ ^149^ ^]^ Lesions developed in PCKS9‐AAV mice have similar morphology and composition to those in LDLr^−/−^ mice but can promote atherosclerosis without a high fat high cholesterol (HFHC) diet.^[^ ^150^ ^]^ The ApoE*3‐Leiden.CETP model displays inflammatory responses and lipid formation like humans; however, there is an absence of plaque rupture, thrombus formation, and/or hemorrhage of the plaque seen in advanced human atherosclerosis.^[^ ^151^ ^]^ Only limited studies in a crossbred ApoE^−/−^Fbn1^C1039G+/−^ model have allowed the study of plaque vulnerability, fibrosis, and SMC apoptosis.^[^ ^152^ ^]^ The Karlheinz Peter double ligation method has been adopted in various mouse models to study the rupture of unstable atherosclerotic plaques, identifying key biological components such as certain epitopes in platelets that may be key diagnosis factors to plaque vulnerability, but plaque calcification and plaque erosion cannot be studied.^[^ ^153^ ^]^ However, despite their utility in studying different stages and aspects of atherosclerosis, mouse models offer limited insight into hemodynamic changes in stenosed arteries.^[^ ^154^ ^]^ The localization of plaque differs in mice compared to humans; plaques usually manifest in the mouse aorta and proximal large vessels relative to small diameter vessels such as coronary arteries in humans ^[^ ^155^ ^]^ and the cardiovascular anatomy and hemodynamics in mice are vastly different to humans. Additionally, the small size of mice limits the assessment of hemodynamic forces such as velocity and pressure waveforms through non‐invasive means using current imaging modalities.^[^ ^156^ ^]^

Several modern imaging techniques, such as micro‐computed tomography (micro‐CT) or magnetic resonance angiography (MRA), have increased spatial resolution and can image mice despite their small stature. Despite this, optimization of settings and post‐processing techniques are required.^[^ ^157^ ^]^ Micro‐CT techniques can identify internal characteristics of the plaque composition and other detailed anatomical structures through non‐invasive means in small rodents.^[^ ^158^ ^]^ The high‐resolution images are achieved by imaging small areas (<200 µm diameter) of the diseased animal.^[^ ^159^ ^]^ Comparatively, MRA can be utilized for a more specific and still non‐invasive method. MRA uses a contrast dye to highlight obstructions in blood flow within arteries and can be used in small animal models once contrast agents and imaging parameters are optimized.^[^ ^160^ ^]^ Higher accuracy in imaging the coronary arteries can also be achieved through an *ex vivo* approach using a high‐resolution synchrotron imaging technique.^[^ ^161^ ^]^ The accuracy may be hindered due to assumptions made while reconstructing the images, and this method cannot explore hemodynamic parameters.^[^ ^162^ ^]^

Like mice, rabbit models are primarily used to study the early stages of atherosclerosis. They can model monocyte adhesion and small lesion formation after several weeks of HFHC diet.^[^ ^163^ ^]^ There are three rabbit models of atherosclerosis, including 1) cholesterol‐fed models, 2) Watanabe‐heritable hyperlipidemic (WHHL) models, and 3) genetically modified models.^[^ ^163^ ^]^ The cholesterol‐fed rabbit models were the first to induce atherosclerosis through an HFHC diet. However, an extended feeding period is required to achieve desirable results, generating concerns over survivability and hepatic toxicity.^[^ ^164^ ^]^ The homozygous WHHL model, which carries a natural defect in the LDL receptor, allows for accelerated atherosclerosis and shows increased levels of LDL and an increased risk of myocardial infarction.^[^ ^164^ ^]^ Conversely, the heterozygous WHHL model exhibits complicated lesions, cholesterol clefts, fibrous caps, and calcification like those in humans.^[^ ^165^ ^]^ WWHL rabbits with coronary lesions have fatty streaks and fibrous plaques, with some containing a lipid core and a thin cap that mimics vulnerable plaque in humans but do not mimic plaque rupture as the mechanical stresses in rabbit vessels are insufficient to trigger rupture.^[^ ^166^ ^]^

Transgenic rabbit models involve one or more human genes involved in atherosclerosis expressed in rabbits. Over twenty different strains have been developed to study the role of lipoproteins, identify susceptibility factors, and understand spontaneous lesion development in atherosclerosis, but are limited in studying advanced atherosclerotic legions.^[^ ^167^ ^]^ A detailed review of these models is available in ref. [168]. Examples include genes coding for Apolipoprotein (Apo) A, ApoA1, ApoA2, ApoB100, ApoE2, ApoE3, hepatic lipase, lipoprotein lipase, and matrix metalloproteinase 12.^[^ ^131^, ^167^ ^]^ Rabbit models are more affordable than large animal models such as pigs and non‐human primates and are closer to humans in terms of lipid metabolism than rodents. However, rabbits lack hepatic lipase, resulting in higher levels of HDL particles.^[^ ^164^ ^]^ Additionally, most rabbit strains are not inbred, resulting in diverse responses to an HFHC diet, limiting reproducibility. Most atherosclerotic lesions in rabbits are found in the aortic branch,^[^ ^163^ ^]^ but when seen in coronary arteries, they have similar features to human atheromas, including the presence of fatty streaks, fibrous tissue, lipid/necrotic core, and fibrous caps.^[^ ^166^, ^169^, ^170^ ^]^ However, the study of stenosed coronary arteries in rabbits is met with the same limitations as rodent models, including low spatial resolution in non‐invasive 3D imaging techniques ^[^ ^171^ ^]^ and deformation of vessels during specimen harvesting.^[^ ^172^ ^]^

Pigs are utilized to study multiple stages and aspects of atherosclerosis due to their large size, allowing for non‐invasive measurements of flow and pressure changes in the presence of atheromas.^[^ ^145^ ^]^ CFD simulations demonstrated no significant differences in intravascular flow features such as near‐wall velocity profiles, time‐averaged WSS, or anatomical features between healthy human and pig coronary arteries, suggesting that pigs are a good representation of the natural physiology in humans.^[^ ^173^ ^]^ The most prevalent pig models are the diabetic/hypercholesteremic swine model, the Rapacz LDL receptor model, and the transgenic D374Y‐PCSK9 pig models.^[^ ^174^ ^]^ HFHC diet in combination with streptozotocin injections (a chemotherapeutic agent that damages pancreatic beta cells resulting in hyperglycemia) is used to induce diabetes^[^ ^175^ ^]^ and atherosclerotic lesions indicative of early‐stage atherosclerosis in pigs.^[^ ^176^ ^]^ However, modeling of late‐stage atherosclerosis requires long feeding periods (≥ 12 months) in standard swine models ^[^ ^177^ ^]^ which poses an additional mortality risk for the animal due to the subsequent significant increase in weight. On the other hand, Rapacz LDL receptor swine models are more prone to higher levels of LDL and lower levels of HDL‐C than diabetic swine models ^[^ ^178^ ^]^ and, thus, may form advanced lesions within 4.5 months of starting an HFHC diet.^[^ ^179^ ^]^ Further, combining an HFHC diet in LDL knockout swine models with balloon injury can reduce the advanced lesion formation timeframe to 3 months.^[^ ^180^ ^]^

Like rabbits, transgenic pig models were created to induce hypercholesterolemia and early development of atherosclerosis similar to humans in a more controlled manner.^[^ ^174^ ^]^ The transgenic D374Y‐PCSK9 model is useful for testing different imaging techniques as lesions form in the aorta, iliofemoral, and coronary arteries at 12 months.^[^ ^181^ ^]^ Overall, these models have exhibited pathological features such as plaque neovascularization, calcification, and foam cell lesions that contain connective tissue and SMCs, which are not present in mouse models. IVUS, OCT, and CTCA imaging demonstrated that pig plaques have similar characteristics to humans, including lipid‐rich necrotic core, calcification, and intraplaque hemorrhage.^[^ ^182^ ^]^ Due to their large size and similar arterial architecture to humans, non‐invasive imaging techniques such as 4D phase‐contrast (PC) MRI can assess velocity profiles and WSS in pigs.^[^ ^183^ ^]^ However, the low spatial resolution of 4D PC MRI in small diameter arteries ^[^ ^184^ ^]^ and the high cost of swine models limit this research.^[^ ^156^ ^]^

Non‐human primate models are considered the most representative of human atherosclerosis as they develop arterial lesions with similar morphology and locations to those seen in humans. However, due to the high cost and level of maintenance, and specialized care required, only limited research has been performed on these models relative to mice, rabbits, or pigs.^[^ ^185^ ^]^ Non‐human primate models have similar lipoprotein patterns exhibiting hypercholesterolemia in HFHC‐fed models,^[^ ^156^ ^]^ plaque morphology, and biochemical characteristics to those seen in humans.^[^ ^186^, ^187^ ^]^ New World monkeys, such as squirrel and Capuchin monkeys, have been used to study atherogenesis and the effects of an HFHC diet, which results in vascular lesions primarily in the aorta with minimal presence in the coronary.^[^ ^188^, ^189^ ^]^ Models developed in Old World monkeys, including African green monkeys and baboons ^[^ ^190^, ^191^ ^]^ exhibit atherosclerosis in coronary arteries and allow the study of psychological factors such as social stress in disease development.^[^ ^192^ ^]^ Atherosclerosis in Old World monkeys was induced through a HFHC diet, similar to the New World monkeys, and due to their large body size, longitudinal studies for advanced atherosclerosis can be conducted.^[^ ^193^, ^194^ ^]^ The large size of non‐human primate models supports non‐invasive imaging and allows more tissue and blood samples to be collected, increasing experimental reliability.^[^ ^145^ ^]^ However, their spatial resolution in small‐diameter vessels and limitations to appropriate radiation exposure restrict imaging studies.^[^ ^195^ ^]^

Due to the complex nature of human atherosclerosis, animal models are critical in developing and testing clinical interventions, but the cost, maintenance, and ethical concerns, as well as differences in anatomy and physiology relative to humans, limit the utility of animal models (**Table** **3**).

**Table 3:** Summary of in vivo models of atherosclerosis and their respective features compared to human atheromas.

		Animal Models			
		Mice	Rabbit	Pigs	Non‐human Primates
Models Studying Specific Stages of Atherosclerosis	Endothelial Dysfunction	ApoE^−/−^	Cholesterol‐fed, WHHL, Transgenic (Human ApoA‐1, ApoA‐2, ApoB‐100, ApoE‐2, ApoE‐3, hepatic lipase, lipoprotein lipase)	Diabetic/hypercholesteremic, Rapacz LDL receptor Transgenic D374Y‐PCSK9	New World Monkeys, Old World Monkeys
	Lipid Metabolism	ApoE^−/−/−^, ApoE*3‐Leiden.CETP, LDLr^−/‐ ‐/−^, PCSK9‐AAV			
	Inflammation	LDLr^−/‐ ‐/−^, PCSK9‐AAV, ApoE^−/−^ Fbn1 ^C1039G+/‐^	Cholesterol‐fed, WHHL, Transgenic (MMP‐12)		
	Fibrosis	ApoE^−/−^ Fbn1 ^C1039G+/−^	Cholesterol‐fed, WHHL		
	Plaque Rupture				
Other Factors Compared to Humans	Plaque Location	In large proximal vessels and aorta	In the aortic branch with limited in coronary arteries	In large and small vessels	In large and small vessels, with a similar predilection to humans
	Plaque Lesion Composition	Low	Low	Moderate	High
	Pathophysiology	Low	Low	Moderate to High	High
	Vessel Wall Stiffening	Present	Present	Present	Present
	Disease Progression	Low	Moderate	High	High
	Imaging of Coronary Artery	Optimized Micro‐CT or MRA	Ultrasound or MRI	Ultrasound, CT, and MRI	Ultrasound, CT, and MRI
	Hemodynamics	Limited	Limited	High	High

### 4.2 In Vitro Models

In vitro models of atherosclerosis are laboratory‐based methods of studying the disease process outside of a living organism. These models can help researchers understand the underlying mechanisms of the disease and develop new treatments. Different in vitro models are used to study atherosclerosis, including 2D models using single or multiple cell types and 3D models using spheroids, microfluidic chips, or tissue‐engineered blood vessels (TEBVs).^[^ ^196^ ^]^

#### 4.2.1 2D Cell Culture

2D cell culture models of atherosclerosis can be broadly categorized into (1) single‐cell cultures, (2) direct co‐cultures, and (3) indirect co‐cultures. Single‐cell cultures are used to study specific cellular processes involved in disease initiation or progression,^[^ ^196^ ^]^ focusing on a single type of cell, such as endothelial cells (ECs), SMCs, or foam cells.^[^ ^197^, ^198^, ^199^, ^200^ ^]^ Researchers observe the effects of different factors on these cells, such as inflammation or drug intervention. For example, adding TNF‐α to a culture of human‐derived ECs can help researchers study the effects of inflammation on the cells.^[^ ^201^ ^]^ These cultures can also be used to test the effectiveness of potential treatments for atherosclerosis. For instance, treating cultured macrophages with IL‐33 reduced the expression of genes involved in cholesterol esterification and thus decreased the chance of foam cell formation.^[^ ^202^ ^]^ Single‐cell cultures are widely used in atherosclerosis research due to their simplicity, cost‐effectiveness, and reproducibility.^[^ ^203^ ^]^ Additionally, advancements in single‐cell sequencing, epigenetics, and cell fate mapping are expected to improve our understanding of atherosclerosis.^[^ ^204^ ^]^ However, single‐cell cultures do not accurately represent the complexity and the cell–cell and cell–matrix interactions in the native 3D tissue environment.^[^ ^205^ ^]^

Co‐culture models that include multiple cell types can reveal the intricate interactions involved in the development of atherosclerosis. Direct co‐culture enables the study of cell–cell interactions, including the exchange of signaling molecules and surface receptor interactions, that cannot be observed in single‐cell cultures.^[^ ^206^ ^]^ On the other hand, indirect co‐cultures where different cell types are separated by a permeable membrane that allows the exchange of nutrients and signaling factors provide a more accurate representation of in vivo conditions.^[^ ^207^ ^]^ Both direct and indirect co‐cultures can be either static or dynamic.

Researchers have utilized static co‐cultures, which do not consider the flow and pulsatile conditions found in physiological systems, primarily to study the biochemical processes involved in atherosclerosis. The most common co‐culture consists of interactions between ECs and SMCs due to their significant role in plaque destabilization and thromboembolic events.^[^ ^208^ ^]^ Previous experiments have shown that ECs play a critical role in the phenotype of SMCs, affecting SMC growth, density, and protein synthesis.^[^ ^209^, ^210^ ^]^ A direct static model of atherogenesis using a co‐culture of rabbit‐derived aortic SMCs, ECs, and monocytes was developed, and human peripheral monocytes were observed to transmigrate and differentiate into macrophages, and foam cells were formed following lipid uptake.^[^ ^211^ ^]^ Static models can also be used within indirect co‐cultures, where for example, a co‐culture of SMCs and monocytes was activated with cytokines derived from T lymphocytes (INF‐γ). The presence of TNF‐alpha andoncastatin M elements from macrophages increased the expression of alkaline phosphatase, which led to an increase in calcification in SMCs.^[^ ^212^ ^]^

In another study, a static three‐cell co‐culture consisting of ECs, SMCs, and foam cells was established to mimic an atherosclerotic plaque microenvironment.^[^ ^213^ ^]^ A therapeutic agent was injected to promote cholesterol efflux and induce foam cell formation, typically seen in the later stages of advanced plaque formation.^[^ ^214^ ^]^ Another approach used a three‐cell 2D culture where ECs and SMCs alongside THP‐1 macrophages showed increased expression of endothelial nitric oxide synthases (eNOS), platelet endothelial cell adhesion molecule 1 (PECAM‐1) and vascular cell adhesion molecule 1 (VCAM‐1). This suggests multidirectional communication between the three cell types during inflammation.^[^ ^215^ ^]^ Models like these contribute to understanding underlying mechanisms involved in atherosclerosis and are expected to aid the development of new treatments for atherosclerosis.

Although static models can shed light on specific biochemical processes involved in atherosclerosis, they do not account for the role of shear stress in the disease.^[^ ^216^ ^]^ Shear stress has been shown to inhibit SMC proliferation and influence ECs’ ability to trigger intracellular signals.^[^ ^217^, ^218^ ^]^ Dynamic models incorporating flow and shear stress are necessary to better mimic physiological conditions. Shear can be induced using a rotating device, such as a disk or a cone. A direct coculture study using a rotating disk demonstrated an increase in the uptake of LDL by cells compared to cultures that were not exposed to external shear flow.^[^ ^219^ ^]^ Also, in an indirect coculture study, a rotating cone was shown to control the shear stress magnitude, thus allowing realistic hemodynamic forces in a 2D layer to be achieved.^[^ ^220^, ^221^ ^]^ However, to make these models more physiologically relevant, the natural architecture of arteries needs to be considered.

Another indirect dynamic co‐culture model is the parallel plate flow chamber (PPFC), where fluid flows between two parallel plates, with the cells on the lower plate.^[^ ^222^ ^]^ Disturbed flow conditions, like those prevalent in stenosed arteries, can be simulated using the cone and plate device and PPFC. However, only laminar fluid flow can be simulated on PPFC.^[^ ^223^ ^]^ Laminar shear flow conditions were simulated in a PPFC with a co‐culture of human umbilical vein endothelial cells (HUVECs) and SMCs, establishing that SMCs regulate the recruitment of leukocytes to ECs.^[^ ^224^ ^]^ Another experiment observed the differences in morphology of ECs and SMCs under laminar conditions in a PPFC, where ECs took on an elongated and parallel to fluid direction morphology, whereas SMCs oriented perpendicularly. Additionally, this study identified the role of shear stress in inhibiting the expression of the intercellular adhesion molecule‐1 (ICAM‐1) and VCAM‐1.^[^ ^225^ ^]^ More detailed reviews of in vitro co‐cultures between SMCs and ECs and of SMC and monocyte/macrophages can be seen in ^[^ ^208^ ^]^ and ^[^ ^226^ ^]^, respectively.

Co‐cultures offer the advantage of better reflecting the diversity and organization of tissues, allowing for the study of complex cellular processes that are difficult to recreate using a monolayer of cells.^[^ ^227^ ^]^ However, experimental results may vary due to differences in cell density, media exchange, and cell type,^[^ ^216^ ^]^ Although single‐cell cultures and direct co‐cultures are useful in studying atherosclerotic processes, hemodynamic forces, and drug development, 2D cell cultures are unsuitable for studying drug uptake and efficacy in complex 3D tissues.^[^ ^228^ ^]^ Therefore, while 2D cell cultures can provide insight into the specific cell phenotypes, they lack the natural architecture critical to model complex processes such as atherosclerosis accurately.

#### 4.2.2 3D Spheroid Cultures

2D cell cultures cannot mimic the 3D microenvironment of blood vessels, have limited cell‐to‐cell interactions, and do not exhibit a diffusion gradient of nutrients or oxygen prevalent in the body^[^ ^229^ ^]^; 3D cell culture techniques are necessary to bridge these gaps. Spheroids are 3D self‐assembled cell aggregates that allow biological interactions relevant to atherosclerosis to be observed.^[^ ^196^, ^230^ ^]^ The most common spheroid formation methods include the hanging drop method, gel embedding, magnetic levitation, spinner culture, and microfluidics.^[^ ^231^ ^]^ A more detailed description of spheroid cell culture and fabrication methods can be found in.^[^ ^232^ ^]^ When cells are placed in the suspension, ECM fibers encourage aggregation, and factors such as E‐cadherin, β‐catenin complex, and actin can create strong adhesive spheroids.^[^ ^231^ ^]^ Despite the diverse phenotypes and ability to mimic the biochemical properties, spheroids can lack reproducibility and are costly and time‐consuming to generate.^[^ ^233^ ^]^

Foam cells are a significant cell type involved in atherogenesis and foam cell spheroids have become a popular model in atherosclerosis research. Foam cell spheroids are primarily used to test the efficacy of anti‐inflammatory drugs such as fluocinolone acetonide (FA) and dexamethasone (Dex). Studies have shown that FA is effective in preventing lipid accumulation and reducing inflammation in foam cells.^[^ ^234^ ^]^ Additionally, drug delivery methods such as targeted ultrasound‐enhanced drug delivery^[^ ^235^ ^]^ or simvastatin‐loaded nano liposomal formulation (LIPOSTAT) can be tested on 3D foam cell spheroids. These methods have demonstrated a reduction in cytokine secretion, monocyte adhesion, and lipid accumulation, indicating the potential of the drug delivery options for inhibiting atheroma formation.^[^ ^236^ ^]^

SMC spheroids can also be used to study atherosclerosis since SMCs promote plaque formation and are directly proportional to plaque stability and vulnerability.^[^ ^237^ ^]^ For example, experiments using a 3D spheroid model composed of SMCs derived from knockout mice fed an HFHC diet over 12 weeks, showed that the growth of atherosclerotic lesions is controlled by membrane type 1 matrix metalloproteinase (MT1‐MMP) expressed by SMCs.^[^ ^238^ ^]^ Further studies used SMC spheroids combined with machine learning to identify changes in morphology through subpopulations or cluster formation when injecting focal adhesion kinase (FAK), Rac, Rho, and Cdc2 inhibitors into the spheroid culture system,^[^ ^239^ ^]^ showing the model's potential for drug treatment testing. Although this model can test the effects of different drug treatments for only one cell type, aggregation of SMCs with ECs in a spheroid model enables researchers to gain a better understanding of atherosclerosis.^[^ ^240^ ^]^ The interaction between SMCs and ECs is critical to atherogenesis ^[^ ^241^ ^]^ and co‐culturing SMCs and ECs in a spheroidal model mimics a blood vessel with a luminal aspect, which increases inter‐endothelial junctional complexes and reduces apoptosis in the ECs due to the presence of SMCs.^[^ ^242^ ^]^

Late‐stage pseudo‐atherosclerotic plaque using myeloid cells has been studied for a more physiologically relevant spheroid plaque model.^[^ ^243^ ^]^ This model encompasses an extracellular matrix (ECM) and can display remodeling aspects lacking in previous spheroid models.^[^ ^243^ ^]^ However, incorporating intimal calcification and T lymphocytes, seen during later stages of plaque development and inflammation, respectively, could further improve the model.^[^ ^244^, ^245^ ^]^

Despite spheroid models exhibiting natural 3D arterial wall architecture, these models cannot demonstrate the mechanical properties of arteries, including the hemodynamic forces experienced,^[^ ^246^ ^]^ which are critical in identifying areas of atherogenesis, plaque rupture events, and plaque phenotype classifications.^[^ ^247^ ^]^ Additionally, spheroid models lack the structure of a coronary vessel, rendering them unsuitable for medical device testing or atherosclerotic growth studies.^[^ ^248^ ^]^

#### 4.2.3 Microfluidic Vascular Devices

Microfluidics uses small volumes of fluids in microchannels to model the cardiovascular system and study pathogenesis and drug treatments.^[^ ^249^ ^]^ Microfluidic chips have multiple microchannels connected to inlet and outlet ports with a pump to control hemodynamic forces. These chips can be made of acrylic, glass, silicon, or PDMS.^[^ ^250^ ^]^ The most common methods of fabricating them are replica molding, embossing, injection molding, or laser ablation.^[^ ^251^ ^]^ Microfluidic devices enable greater control over experimental conditions because the customized design can replicate complex biological processes such as atherosclerosis. It also reduces the time taken for experiments and offers flexibility in application due to the compactness and versatility of the device.^[^ ^252^ ^]^ A review detailing the different types of microfluidic chips and their advantages and disadvantages can be found in ref. [253].

Microfluidic chips are often used to investigate the different stages of atherosclerosis. These chips can be made of PDMS and can even include pneumatic actuation of a membrane to create different levels of stenosis severity.^[^ ^254^ ^]^ In one model, a low‐level occlusion resulted in high shear stress at the stenosis region, while an 80% occlusion produced low shear regions at the apex of the stenosis, observations validated through fluid simulation and other experimental studies. Low shear stress areas were also associated with increased monocyte adhesion, consistent with previous in vivo studies. However, these microfluidic devices do not accurately model complex fluid profiles such as pulsatile flow and the atherosclerotic microenvironment required for foam cell formation and ECM remodeling. To address this, a modified surface tension‐based ECM patterning method has been developed and allows for the creation of an intima‐media‐like structure mimicking the physiological ECM composition.^[^ ^255^ ^]^ Early atherogenic events can be observed by seeding SMC and ECs and introducing cytokines and oxLDL proteins. This has been shown to increase SMC migration and monocyte‐to‐EC adhesion, both of which are indicators of inflammation.

One study used a microfluidic device to simulate “fatty streak” atherosclerosis by coating hydrogels with fibronectin and seeding them with ECs.^[^ ^256^ ^]^ This increased cell permeability, leading to SMC proliferation and the disruption of endothelial barrier integrity, but the device could not detect functional remodeling, a critical aspect of understanding atherosclerotic morphology.^[^ ^257^ ^]^ Another study created a two‐layered engineered vascular graft by seeding ECs and SMCs on a polyglycolic acid (PGA) scaffold and inducing atherogenesis through a custom fluidic device. By adding iPSC‐derived macrophages and LDL concentration, the experiment produced transcriptional levels of genes for ECM assembly and remodeling like those found in native plaques.^[^ ^258^ ^]^ The experiment was compared to CFD models incorporating lipid accumulation and fluid flow, identifying atheroprone and high‐lipid‐accumulated in both computational and experimental settings. However, the age and sex of cells were not considered despite atherosclerotic plaques being found in fetuses,^[^ ^259^ ^]^ children,^[^ ^260^ ^]^ young adults,^[^ ^261^ ^]^ and elderly subjects.^[^ ^262^ ^]^ T and B cells, which have been shown to affect early atherogenesis, were omitted due to methodological constraints.^[^ ^245^ ^]^

WSS and cyclic strain are two hemodynamic factors associated with atheroprone locations and plaque formation.^[^ ^263^ ^]^ To study these factors, microfluidic devices have been developed that can expose cultured cells to physiological levels of fluid shear stress and cyclic stretch.^[^ ^264^ ^]^ In one study, four different hemodynamic profiles were simulated, including a control group, tachycardia, disturbed flow, and atherosclerotic conditions, which found that abnormal fluid shear stress values and increased levels of reactive oxygen species led to morphological changes in vascular cells similar to in vivo experiments.^[^ ^265^, ^266^ ^]^ Another experiment used a gelatin‐based model to investigate the effects of WSS on the endothelial layer. By identifying areas of laminar and turbulent flow using CFD models, researchers found that low WSS resulted in a rounded and disordered arrangement of ECs, indicating susceptibility to plaque formation. Additionally, increased expression of cellular adhesion molecules (ICAM‐1 and VCAM‐1) critical to atherogenesis was found in this location, and a similar trend was observed in SMCs of human atherosclerotic plaques.^[^ ^267^ ^]^ A plaque‐on‐a‐chip model was designed to observe hemodynamics in atheroprone conditions.^[^ ^268^ ^]^ Validation through CFD analysis identified analogous regions of inflammation and areas of LDL accumulation, particularly in low WSS regions.

While many of the methods discussed above aim to understand the factors contributing to atherogenesis, there are microfluidic devices specifically designed to study atherothrombosis.^[^ ^269^ ^]^ One such device uses a gelatine‐collagen composite hydrogel to simulate various degrees of stenosis and found that increased turbulence and shear stress led to more platelet attachment and faster occlusion.^[^ ^269^ ^]^ Moreover, these devices allow researchers to test the effectiveness of drugs like aspirin. Other studies have also focused on analyzing the differences in platelet thrombus formation in the presence or absence of antithrombotic agents and studying the effects of shear rates to better understand the efficacy of antiplatelet therapies in preventing occlusive conditions.^[^ ^270^, ^271^ ^]^ Another study described the need for organ complexity required to adequately represent the current physiological mechanisms of thrombosis and how microfluidic chips can aid in the generation of such models via increased resolution and assembly‐free manufacturing.^[^ ^272^ ^]^ Although microfluidic chips can be used for diagnostics and to enhance our understanding of atherosclerosis, they can also be used for pharmaceutical evaluations. One study utilized the process of photolithography to generate PDMS microfluidic chips to test the efficacy of different anti‐thrombotic drugs.^[^ ^273^ ^]^ A monolayer of HUVECs was established and TNF‐α and abciximab was added to induce endothelial dysfunction and affect platelet function, respectively. This method discerned a clear difference between patients of healthy and diseased status.

Microfluidic devices offer precise control and manipulation of fluid flow,^[^ ^274^ ^]^ allowing replication of complex flow patterns and physiological conditions relevant to atherosclerosis. Blood flow changes in stenosed arteries are studied by allowing for the analysis of hemodynamic forces and mass transport of nutrients through fluid flow.^[^ ^275^ ^]^ They also integrate real‐time monitoring capabilities, thus enabling observation of cellular behavior, molecular interactions, and disease progression in real time.^[^ ^276^ ^]^ Differences in cell numbers, media turnover due to increased glucose consumption, reduced proliferation, and pH for regulation between normal 3D cell cultures and microfluidic chips have also been observed and need to be noted.^[^ ^252^ ^]^ Additionally, microfluidic chips are limited in their ability to scale to large numbers and be incorporated into medical device testing due to their small size and specificity in design.^[^ ^277^ ^]^ Despite the tunability of microfluidic chips to create bifurcations, curvatures, and stenoses in the channels,^[^ ^278^ ^]^ the rigidity and rectangular shape of the channels do not mimic the viscoelastic nature and physiological geometry of native blood vessels, respectively.

#### 4.2.4 Tissue‐Engineered and Biofabricated 3D Models

Tissue engineering and biofabrication are multidisciplinary research fields integrating principles from biology, engineering, and medicine to create functional biological tissues with the aim of regeneration or replacement of damaged/diseased tissue or to serve as in vitro tissue or diseased models.^[^ ^279^, ^280^ ^]^ Advances in these research areas have made it possible to recreate the hierarchical nature of blood vessels, mimic the stenosed vessel systems, and make intricate and customizable 3D in vitro models.^[^ ^280^ ^]^ The use of tissue engineering and biofabrication in the context of modeling atherosclerosis is a relatively new field, but key studies are demonstrating the utility of these technologies in establishing critical aspects of stenosed vessel anatomy and pathophysiology (**Table** **4**).

**Table 4:** Tissue engineered (TE) and biofabricated blood vessels in atherosclerosis studies.

Methodology	Blood Vessel Material	Cells Used	Atherosclerosis Stages Simulated				Induction of Atherosclerosis	Validation	Ref
			Endothelial Dysfunction	Fatty Streak Formation	Plaque Growth/ Stenosis	Plaque Rupture/ Erosion			
Commercially sourced	PGLA, ePTFE	N/A	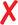	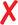	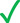	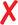	Curvature of geometry (S/L/U‐shaped)	N/A	[309]
Heat welding	PGA‐P4HB (poly‐4‐hydroxybutyrate)	HUVECs, human umbilical cord derived myofibroblasts (UCMFBs), human monocytes	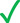	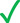	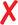	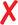	LDLs and HDLs	N/A	[305]
Commercially sourced	ePTFE	HUVECs	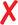	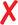	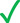	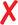	Curvature of geometry (S/L/U‐shaped), incorporation of stent	N/A	[310]
Support bath coaxial bioprinting	Vascular tissue‐derived decellularized ECM bioink	HUVECs, HCASMCs, HDFs	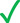	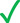	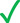	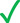	Changes in geometry of blood vessel (stenosis of ∼25% of bending of ∼150°) with cell seeding, introduction of TNF‐α and human LDLs	N/A	[303]
3D Printing, Mold Casting	Ferroelectric potassium sodium niobate, gerroelectric polyvinylidene fluoride, PDMS	N/A	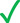	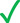	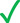	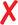	Changes in geometry of blood vessel (0‐80% stenosis)	N/A	[302]
Orbital Shaker Dish Platform	PGA‐P4HB	SMCs, Have Collagen, Elastin	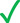	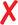	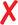	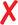	Introduced high shear stresses	CFD simulations	[315]
Fabrication Molds	Collagen	HUVECs, Human coronary artery SMCs (hCASMCs), human monocytes, human neonatal dermal fibroblasts (hNDFs)	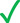	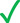	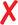	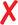	Introduced eLDLs and TNF‐α	N/A	[146]
Gel‐Molding	Collagen	HUVECs, human monocytes	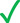	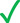	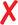	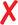	Branched geometry of blood vessel (45°, 60° and 80° bent), introduced eLDLs and TNF‐α	PIV experiments, CFD simulations	[311]
Volumetric Bioprinting, Melt Electrowriting	Medical grade polycaprolactone, gelatin methacryloyl	Human mesenchymal stromal cells (hMSCs), HUVECs,	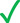	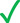	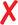	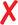	Changes in blood vessel geometry (∼50% stenosis, eccentric and concentric)	N/A	[316]

In tissue‐engineering and biofabrication, blood vessels are typically made by interfacing ECs, SMCs, and/or fibroblasts with biomaterials to create hierarchical tubular constructs that can be connected to a bioreactor to replicate or measure physiological factors such as shear stress, shear strain, pulsatile flow, and blood pressure.^[^ ^281^ ^]^ Material choice in these systems is based on the ability to recapitulate the biological and mechanical features of native vessels, such as burst pressure, tensile strength, compliance, porosity, hemocompatibility, and cell types supported.^[^ ^282^ ^]^ A range of materials have been tested including synthetic polymer grafts (Dacron and ePTFE),^[^ ^283^, ^284^, ^285^ ^]^ biodegradable synthetic polymer grafts (PLGA and PCL),^[^ ^286^, ^287^, ^288^ ^]^ and natural polymer grafts.^[^ ^289^, ^290^, ^291^, ^292^, ^293^, ^294^ ^]^ Biodegradable synthetic grafts have favorable mechanical properties and can support endothelialization,^[^ ^295^ ^]^ but regression of cell adhesion and thrombogenicity are seen in long‐term studies.^[^ ^296^ ^]^ Conversely, natural polymer grafts, such as those made from collagen, elastin, or silk fibroin, are more hemocompatible and support endothelialization and growth of SMCs; however, they lack physiological compliance.^[^ ^282^ ^]^ In biomaterial‐free approaches, a series of studies generated tissue‐engineered blood vessels (TEBVs) by rolling sheets of cells around a cylindrical mandrel, relying entirely on the ECM produced by the cells for structural integrity.^[^ ^280^ ^]^

The fabrication technique plays a crucial role in tuning the mechanical properties of biomaterials, and therefore should be considered. A detailed review of these materials with differing fabrication techniques can be found in.^[^ ^282^, ^297^, ^298^ ^]^ Advances in fabrication techniques, such as the introduction of 3D bioprinting, allow rapid precise deposition of bioinks, a combination of cells and biomaterial, to generate tubular constructs and mimic stenosed vessels (Table 4). In addition to making the constructs, bioreactor design is critical to further improve the fabricated modes by applying mechanical forces such as cyclic stretching or pulsatile flow. This enables control of the tissue microenvironment and helps monitor the growth and development of the construct.^[^ ^294^, ^299^ ^]^ One of the major challenges with tissue‐engineered and biofabricated tissue models is the flow visualization through the hierarchical 3D construct. A study on bioreactor designs developed a system to incorporate live cell‐to‐cell interactions and real‐time monitoring of cellular dynamics using 3D confocal microscopy and post‐analysis using computer vision, but these technologies have not yet been utilized in atherosclerotic studies.^[^ ^300^, ^301^ ^]^

Although there is only a limited number of models that take into account vessel stenosis, the work to date demonstrates distinct advantages of hierarchical 3D models over 2D models and demonstrates the potential of these technologies in enhancing our understanding of atherosclerosis. One study utilized ferromagnetic inks, supplemented with functionalized sodium potassium niobate as piezoceramic particles, to produce a sinusoidal printed artificial artery with pressure‐sensing abilities.^[^ ^302^ ^]^ The piezoelectric voltage records differences in pressure in systole and diastole and can be transferred into stenosed models to monitor graft occlusion by the increase in pressure. Co‐axial 3D printing is used to create straight, tortuous, and stenosed tubular structures from vascular tissue‐derived decellularized ECM, which allowed for endothelialization, growth of SMCs, and incorporation of fibroblasts, recreating an intricate atherosclerotic model of the coronary artery.^[^ ^303^ ^]^ However, these scaffolds lacked tensile strength and exhibited unstable structural integrity. In another study, 3D printing was used to micro‐engineer 3D stenosis models featuring 3D microchannels with different degrees of constriction.^[^ ^304^ ^]^ This enabled the investigation of the anti‐thrombotic and immunomodulatory effects of aspirin and metformin, allowing for multiple analyses of critical atherogenic events such as endothelial dysfunction and platelet and leukocyte adhesion. However, the reduction in vessel size and rigidity in vessel geometry limits its applicability for medical device testing and representation of in vivo conditions.

To study atherogenesis, a polyglycolic‐acid (PGA) tubular scaffold was seeded with HUVECs, and fluorescent LDL, HDL, and labeled monocytes were injected into the bioreactor circulation loop and tracked.^[^ ^305^ ^]^ The results showed a higher level of monocyte adhesion to the endothelial layer when LDLs and TNF‐α were introduced, which indicates the utility of this model in capturing features of atherogenesis. Histological analysis revealed a microstructure similar to that of native arteries and to that of previous in vivo studies. However, further experiments are required to validate the hemodynamics represented within the graft since atherosclerotic lesions typically develop at the site of altered blood flow.^[^ ^306^ ^]^ Although umbilical cord‐derived cells are cost‐effective, coronary‐sourced cells are crucial in assessing many intravascular therapies due to their site‐specificity and should be incorporated in future studies.^[^ ^307^ ^]^

In a similar experiment, a diseased model was created by introducing modified LDL, TNF‐α, macrophages, and monocytes to three‐layered models made by interfacing ECs, SMCs, and fibroblasts with a collagen scaffold.^[^ ^146^ ^]^ Monocyte adhesion was not observed under healthy conditions, but under diseased conditions, increased monocyte adhesion and transmigration of media were observed, resulting in the formation of foam cells. Although the model was only 0.6 mm in luminal diameter, it included a vascular chip that can control various clinical events, making it suitable for drug testing. However, the shear stress exhibited within the model is not indicative of in vivo conditions within human coronary arteries due to the small scale of the vessels.

As early studies have shown that low shear stress is more prevalent in branched vessels and does impact the epidemiology of atherosclerosis^[^ ^308^ ^]^ synthetic tubular constructs in S‐, L‐, and U‐shaped geometries were fabricated and analyzed to investigate the effects of vessel curvature. The experiments showed that strong curvature leads to high shear stress values and ultimately to high cell death.^[^ ^309^, ^310^ ^]^ More anatomical 3D models aim to investigate the impact of branching angles on early atherosclerosis formation. Branched models with angles of 45°, 60°, and 80° were developed and treated with enzyme‐modified LDLs, and TNF‐α to induce EC dysfunction and monocyte adhesion.^[^ ^311^ ^]^ Particle image velocimetry was employed to obtain flow profiles at the inlet and the walls of the two outlets in the branched models. Monocyte adhesion and foam cell formation locations coincided with low WSS locations in PIV and CFD simulations. However, physiologically relevant blood flow values were not achieved in this study. The CFD simulations used for validation also did not consider the elastic properties of the system and assumed a Newtonian fluid with a laminar flow profile. Moreover, a sinuous function was used to represent the pulsatile nature of blood flow, which may not accurately reflect the coronary blood flow profile and unveil further improvements for these models.

Taken together, advanced 3D models have several advantages for studying atherosclerosis. They can replicate physiological conditions and provide a more controlled experimental environment than animal models or clinical studies, allowing for precise measurements and manipulation of variables.^[^ ^312^ ^]^ Moreover, these models are beneficial for ethical reasons as they reduce the need for animal models. However, the simplicity of the in vitro environment limits the ability to translate findings to the human body, and the lack of standardization in the field can affect the reliability and reproducibility of results.^[^ ^313^ ^]^ Improvements in physiological relevance by incorporating more cell types, such as immune cells, and developing more advanced bioreactor systems can better replicate in vivo hemodynamic conditions.^[^ ^281^ ^]^ This will allow the field to move beyond the early promising studies into atherogenesis to studying advanced atherosclerosis models.^[^ ^196^ ^]^ Furthermore, incorporating patient‐specific cells and tissues into blood vessel models would enable more personalized disease modeling and drug screening.^[^ ^314^ ^]^

## 5 Critical Outlook and Future Directions

The discourse above describes computational and biological models of atherosclerosis and highlights their unique advantages and differences between them. While computational models offer benefits in terms of time, cost, and control of experimental variables, they fall short in terms of biological complexity. As a result, the combination of both types of models can harness their unique features to create a more comprehensive understanding of atherosclerosis. Importantly, advancements in this domain necessitate an integrated multidisciplinary approach, drawing expertise from both researchers and clinicians proficient in the diagnosis and treatment of atherosclerosis, as well as in its biological and computational modeling.

### 5.1 Combination of In Silico and In Vitro Experiments

In silico and in vitro experiments have advantages and disadvantages that make them complementary approaches to modeling atherosclerosis. Computational models of hemodynamic vessels are efficient, cost‐effective, and highly controllable but lack biological complexity. Due to the complexity of the cardiovascular system, specific cell–cell and cell–ECM interactions are challenging to express in an equation, and extensive computational memory, time, and possibly more complex software render the simulation inefficient and ineffective. Combining both types of experiments would eliminate these disadvantages, improving accuracy, efficiency, and understanding of the underlying mechanisms of atherosclerosis while creating a pathway for facilitating the translation of the research into potential drug targets or therapies more efficiently. The computational model can provide predicted outcomes for specific conditions which can then be tested experimentally allowing not only validation of the computer model but also providing novel biological insights and reducing developmental time from biological experiments. The computational model itself can also provide insight into the temporal dynamics of different biological processes, serving as a cost‐effective and ethically responsible alternative.

Several studies have combined in silico and in vitro experiments in other disease models,^[^ ^317^, ^318^ ^]^ but this is yet to be demonstrated for atherosclerosis. There have been many instances of combining biological and computational modeling, especially in the microfluidics field, whereby validation of in silico models can be achieved, suggesting a need for a combined approach.^[^ ^267^, ^268^ ^]^ Further, in FSI computational models, researchers are developing biomaterial models to capture the hyperelastic and deformative nature of biological materials, such as coronary vessels and plaque. The challenge lies in accurately modeling distinct structures due to the variation in their biomechanics.^[^ ^319^ ^]^ In vitro and ex vivo experiments with biomaterials and human explants are, therefore, essential for obtaining accurate information to refine computational simulations. Computational models can optimize in vitro experiments and reduce the need for manual optimization of experimental conditions, while in vitro experiments can validate in silico predictions for a more comprehensive analysis.

### 5.2 Validation of Models

Validation is critical for both in silico and in vitro experiments to ensure that the model has enough rigor to sufficiently represent the physiological features of atherosclerotic arteries.^[^ ^320^ ^]^ CFD and FSI experiments have been validated against in vitro experiments, but computational models are rarely used to inform the design or to validate in vitro models in this context. However, typical in vitro experiments used to validate computational simulations often lack biological components due to the researchers’ limited expertise or increased time and complexity. Prospective studies that integrate biological components to mimic the mechanical properties of the arterial wall and plaque would offer a more robust methodology for comparing computational and in vitro data sets. Subsequent computer simulations on the transport of biological agents could be authenticated by validating them against biological perfusion loops. Further, clinical data should be included in model development and validation to truly capture the complexity of the human vessel anatomy and physiology. Clinical data allows for the identification of differences between research findings and clinical practice, which can inform the development of new diagnostic tools, therapies, and prevention strategies. Patient images, blood flow, and pressure waveforms are critical clinical data for assessing stenosed coronary arteries' hemodynamics, especially when reproduced in in vitro experiments. However, clinical data for model validation is very scarce due to limited availability, patient confidentiality, time constraints in obtaining data, and inter‐patient variability.^[^ ^321^ ^]^ Improved interdisciplinary collaborations between medical researchers, biomedical engineers, and clinicians are vital to overcoming access barriers to clinical data.

### 5.3 Advances in Computational and Imaging Technology

Computational modeling has been instrumental in improving our understanding of the various stages of atherosclerosis, such as atherogenesis, plaque progression, and plaque rupture. Specifically, computational models enable the analysis of pressure, flow, and shear stress experienced in stenosed coronary arteries. However, as the simulation becomes more complex due to irregular geometry, fine meshing, and the inclusion of mechanical properties, there is a corresponding increase in computational cost and time. To reduce this computational burden, researchers typically employ techniques such as mesh optimization, model simplification, parallel computing, or the use of a high‐performance computer. However, these approaches may not always be practical for complex atherosclerotic systems or accessible to researchers. Nonetheless, computer architecture and cloud computing advances have significantly increased processing power and access to large‐scale computing resources. Moreover, improvements in algorithms and software, such as the incorporation of machine learning and AI,^[^ ^322^ ^]^ have facilitated the development of more efficient and accurate simulations, making it possible to model larger and more complex systems.^[^ ^323^ ^]^ Another novel technology is quantum computing, which can overcome the current limitations of computing, where biomolecular interactions, molecular dynamics, and complex biological networks during atherosclerotic events can be computed, providing a more detailed visualization of the processes involved in atherosclerosis.^[^ ^324^ ^]^ These advancements have broadened the scope and capabilities of computational modeling for studying atherosclerosis, providing researchers with powerful tools for investigating the disease.

One of the critical limitations of the current models of atherosclerosis is the inability to accurately replicate the physiological blood circulation due to limited data on microcirculatory resistance and other physiological variables, mainly due to limitations in cardiovascular imaging techniques.^[^ ^273^ ^]^ CCTA, IVUS, OCT, and MRI have greatly improved the ability to detect, diagnose, and treat coronary artery disease in patients. However, optimizing image quality and resolution remains an active area of research.^[^ ^274^ ^]^ For example, atheroma geometry is currently only visible through IVUS imaging, but imaging artifacts and poor image resolution affect the reconstruction of the plaque geometry. Dual‐energy CT techniques have demonstrated better image quality and luminal assessment of the coronary artery. This allows for enhanced characterization of atherosclerotic plaques and evaluation of myocardial ischemia compared to single‐energy CT scans.^[^ ^276^ ^]^ However, limited availability and lack of clinical validation hinder the widespread use of this technique. Despite this, dual‐energy CT paved the way for more advanced technologies, such as multi‐energy CT scanners and photon‐counting CT scanners equipped with improved resolution and image quality. Researchers also utilize machine learning and AI techniques to improve image processing and reconstruction to evaluate coronary flow patterns.^[^ ^275^ ^]^ Improved imaging will provide more accurate anatomical and physiological data for the computational and biological modeling of atherosclerosis.

### 5.4 Advances in Fabrication of Functional 3D Models

Early‐stage atherosclerotic models provide valuable insights into the initial stages of plaque formation by investigating changes in endothelial function, lipid accumulation, and immune cell infiltration.^[^ ^305^ ^]^ However, late‐stage atherosclerotic models simulate advanced plaque formation, arterial narrowing, and plaque rupture,^[^ ^243^, ^325^ ^]^ which are responsible for severe disease complications such as myocardial infarction and stroke.^[^ ^304^ ^]^ These models will significantly contribute to developing novel therapeutic strategies to stabilize vulnerable plaques and prevent life‐threatening cardiovascular events. Despite this, minimal research has been conducted on late‐stage atherosclerotic models.

Tissue engineering and biofabrication are rapidly advancing fields at the intersection of biology and engineering and hold great promise in developing late‐stage atherosclerotic models. By integrating advanced fabrication techniques such as 3D bioprinting, intricate and physiologically relevant models that mimic complex geometries and the microenvironment of late‐stage atherosclerotic arteries can be created. These models can incorporate multiple cell types and extracellular matrix components ^[^ ^146^ ^]^ to accurately recreate the cellular composition and architecture of diseased arteries. Advanced fabrication techniques allow for the precise control of spatial organization, cell‐to‐cell interaction, and mechanical properties,^[^ ^326^ ^]^ facilitating the study of critical factors influencing plaque stability and rupture,^[^ ^327^ ^]^ such as cellular components like SMCs ^[^ ^328^ ^]^ or altered hemodynamics.^[^ ^329^ ^]^ They can also be used to investigate the relationship between different cell populations, altered hemodynamics, matrix remodeling, inflammatory response, and mechanical forces acting on the atheroma.

Incorporating CFD modeling can further enhance the utility of advanced fabrication techniques, especially for late‐stage atherosclerotic models. CFD simulations can provide insights into hemodynamic characteristics within the printed vascular structures, such as fluid velocity, pressure distribution, and WSS. Combining patient‐specific cells and geometries, and CFD modeling could offer a robust framework for developing late‐stage atherosclerosis models that closely resemble the complex vascular environment. It enables the study of the intricate interplay between biomechanical forces, local hemodynamics, and plaque vulnerability. Such models could also provide the platform to explore the effects of different mechanical stimuli on plaque rupture, thrombosis, and emboli formation and facilitate the evaluation of novel interventions, such as stents, drug delivery systems, and tissue‐engineered approaches, in a more physiologically relevant context.

The next frontier in tissue engineering and biofabrication are technologies to interface multiple fabrication methods to adequately capture the anatomical, mechanical, and physiological complexity of stenosed vessels by combining different materials and material formats. For example, one of the key challenges is the generation of hierarchical perfusion networks where larger vessels perfuse smaller conduits, an undertaking that is not easily solved with the current technologies. To achieve this, a combination of different fabrication technologies is required to create constructs that simulate the complex flow patterns within branching pathways associated with atherosclerotic growth.^[^ ^330^ ^]^ This requires the development and integration of advanced fabrication systems to model the full complexity of atherosclerosis.^[^ ^331^, ^332^ ^]^ A recent study demonstrated the multimodal fabrication principle in atherosclerosis by combining volumetric bioprinting with melt electrowriting to create stenosed vessels with physiologically relevant mechanical properties.^[^ ^316^ ^]^ Multimodal approaches will allow tuning of the vessel properties to match the changes in the mechanical properties of the vessel wall and plaque associated with atherosclerosis progression, ultimately improving the utility of tissue‐engineered and biofabricated models.

### 5.5 Advances in Single‐Cell Omics Technology

In addition to developing robust models that capture the anatomical and physiological complexity of stenosed blood vessels, techniques to analyze the effects of different variables on cellular responses in these models are equally important for model refinement, validation, and adoption in medical and clinical research. Omics‐based techniques are increasingly used to analyze biological and pathological systems because they capture extensive biological complexity in a single experiment. Omics data encompasses large‐scale biological information generated through various techniques, such as genomics, transcriptomics, proteomics, and peptidomics, offering insights into the functions, interactions, and roles of different molecules in biological processes.^[^ ^333^ ^]^ Single‐cell RNA sequencing has been extensively employed to study diverse aspects of atherosclerosis, including cellular composition in human and mouse atheromas, biochemical signaling between cells, and cellular changes in different sex or hemodynamics conditions.^[^ ^334^ ^]^ One study delved further into the microanatomy of atherosclerotic plaques, providing a transcriptome‐based cellular landscape of the atheromas and identifying intercellular communications that drive inflammation at the plaque site.^[^ ^335^ ^]^

However, a significant challenge in single‐cell transcriptomics lies in the loss of spatial information, which is vital for understanding the mechanisms of atherosclerosis disease and interpreting cell‐to‐cell communication.^[^ ^334^ ^]^ Advancements in spatial sequencing technology have enabled sequencing at individual cell resolution.^[^ ^336^ ^]^ For instance, a recent study utilized Visium spatial transcriptomics technology on six plaques, identifying higher expression of matrix metallopeptidase 9 (MMP9) in unstable or ruptured plaques and associating higher MMP9 levels with the risk of developing coronary atherosclerosis.^[^ ^337^ ^]^ While changes in gene expression related to atherosclerosis can be observed, effectively translating this knowledge into treatments remains challenging.^[^ ^338^ ^]^

Analyzing only one type of omics data does not allow for an in‐depth dissection of the complex processes involved in atherosclerosis. An investigation into the molecular mechanisms of plaque vulnerability and rupture utilized transcriptomics, proteomics, and peptidomics of 42 patient‐derived samples.^[^ ^339^ ^]^ The comparison between single‐omics and multi‐omics data revealed similar results in distinguishing gene expressions between non‐vulnerable and vulnerable plaques. However, the multi‐omics data showed more evident processes before and after plaque rupture. To strengthen these findings further, incorporating more diversity by considering both male and female participants and including metabolomics and lipidomics data is important.^[^ ^340^, ^341^ ^]^

With the increasing output of omics data, integrating different omics datasets with molecular dynamics simulations, CFD simulations, or AI is becoming increasingly important to capture the interplay between biochemical and biomechanical factors involved in disease progression.^[^ ^342^ ^]^ Machine learning has previously been applied to transcriptomics for diagnostic studies of liver injury and mental illness based on gene expression data, but its application in atherosclerosis remains limited.^[^ ^342^ ^]^ Recently, the development of virtual transcriptomics, combining machine learning with CTCA images of stenosed carotid arteries and transcriptomics, demonstrated molecular signatures of the plaques, showing potential for patient‐specific therapies.^[^ ^343^ ^]^ Another study utilized multi‐omics (transcriptomic, genomics, nuclear magnetic resonance, metabolomics, and lipidomics) along with AI strategies to identify preventative markers and provide an unbiased assessment of the pathophysiological correlations in atherosclerosis, allowing for efficient analysis of large‐scale omics data.^[^ ^344^ ^]^ Thus, the combination of AI or simulations with multi‐omics allows for a comprehensive understanding of molecular complexities to enhance biomarker discovery, facilitate predictive disease modeling, and accelerate drug development. Achieving these advancements will depend on fostering synergistic relationships between bioinformaticians, biomedical engineers, and clinicians.^[^ ^345^ ^]^

## 6 Conclusion

Computational modeling has become a popular tool for analyzing complex biological processes such as atherosclerosis due to its flexibility in testing different physiological conditions and ability to analyze isolated hemodynamic parameters. Current computational models of atherosclerosis are increasingly complex, exploring interactions between blood, the vessel wall, and plaque. Yet, validation against the models used in in vitro experimentation or clinical data remains limited. Additionally, biological interactions between cells and agents involved in atherosclerosis cannot be simulated due to the high computational cost of time and high expense of the software and hardware involved. On the other hand, aspects of atherosclerosis initiation and disease progression are captured in a variety of biological models, ranging from simple 2D cell cultures to large animal models. Individually, no model can capture all the complexity of atherosclerosis, but advances in imaging and fabrication are driving the development of increasingly sophisticated 3D patient‐specific computational and biological models of atherosclerosis. The next step for the field is to effectively integrate these technologies for model refinement, validation, and application in discovery research, diagnostics, and drug and medical device development and testing. Omics technologies and tools for big data analysis and integration such as machine learning and AI are making these multi‐scale multi‐modal approaches increasingly accessible, but this progress must be driven by truly multidisciplinary interactions between researchers, engineers, and clinicians.

## Conflict of Interest

The authors declare no conflict of interest.
